# Iran's *Escherichia coli* AMR Landscape (2010–2025): A One Health Systematic Review and Meta‐Analysis

**DOI:** 10.1002/mbo3.70420

**Published:** 2026-10-04

**Authors:** Arezoo Dehghan, Mina Arabian, Zahra Tahmasebi, Aliasghar Fakhri‐Demeshghieh, Neda Baseri, Ehsan Mostafavi, Farzad Badmasti, Faranak Abnaroodhelleh, Arash Seifi, Fahimeh Bagheri Amiri

**Affiliations:** ^1^ Department of Epidemiology and Biostatistics, WHO Collaborating Center for Vector‐Borne Diseases, Research Centre for Emerging and Reemerging Infectious Diseases Pasteur Institute of Iran Tehran Iran; ^2^ Department of Bacteriology, Faculty of Medical Sciences Tarbiat Modares University Tehran Iran; ^3^ Department of Bacteriology Pasteur Institute of Iran Tehran Iran; ^4^ Head of the Control and Supervision Group on the Production of Medicines and Biological Materials of the Iran Veterinary Organization Tehran Iran; ^5^ Center for Communicable Diseases Control (CDC) Ministry of Health and Medical Education Tehran Iran; ^6^ Research Center for Antibiotic Stewardship and Antimicrobial Resistance, Infectious Diseases Department, Imam Khomeini Hospital Complex Tehran University of Medical Sciences Tehran Iran

**Keywords:** antimicrobial resistance, *Escherichia coli*, Iran, one health

## Abstract

*Escherichia coli* (*E. coli*) has developed resistance to multiple antibiotic classes, complicating treatment in both human and veterinary medicine. This study assesses the prevalence of antimicrobial resistance and antimicrobial resistance genes in *E. coli* in Iran, within a One Health framework encompassing humans, animals, foods, and the environment. We systematically searched Scopus, Google Scholar, Magiran, PubMed, Scientific Information Database Library (SID), and Web of Science, using keywords related to AMR and *Escherichia coli*, restricting to English and Persian publications from 2010 to January 2025. The risk of bias was evaluated using the JBI checklist for prevalence studies. Pooled prevalence and 95% confidence intervals (CIs) were calculated for each antibiotic using random‐effects models. A total of 368 studies were included in the meta‐analysis. The highest resistance was observed in the Classes of Penicillins (71.7, 95% CI: 65.6–76.6) and the lowest for Fosfomycins (6.8, 95% CI: 5.2–8.5). Resistance to Ceftizoxime, Ertapenem, Streptomycin, Piperacillin‐Tazobactam, and Colistin increased over time. Phenotypically, 47.2%, 20.2%, and 14.1% of *E. coli* isolates were identified as extended‐spectrum β‐lactamase‐producing (ESBL), AmpC, and metallo‐beta‐lactamase (MBL) producing, respectively. Among ESBL‐producing isolates, the prevalence of *bla*_CTX‐M_, *bla*_TEM_, and *bla*_SHV_ genes was 68.2%, 58.5%, and 24.4%, respectively. The high prevalence of AMR highlights the critical need for robust antimicrobial stewardship within a One Health framework to preserve antibiotic efficacy. Our study provides essential baseline data across human, animal, and environmental sectors in Iran, forming a basis for future One Health strategies and policy‐making.

## Graphical abstract

This One Health systematic review and meta‐analysis of 368 studies (2010–2025) reveals that 47.2% of *E. coli* isolates in Iran are ESBL‐producers, with alarming resistance to penicillins, cephalosporins, and fluoroquinolones. Critically, resistance to last‐resort antibiotics—colistin and ertapenem—is significantly increasing over time.

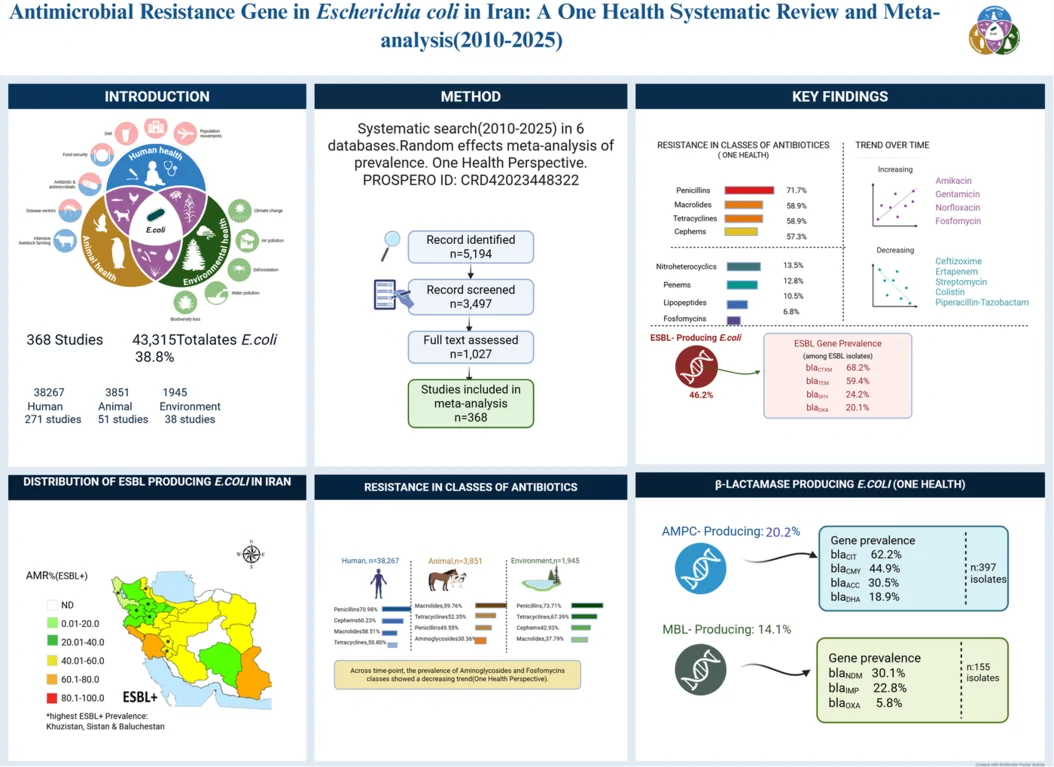

## 1 Introduction

Antimicrobial resistance (AMR) is now recognized as one of the greatest global public health threats, driven by interconnected human, animal, and environmental factors. The World Health Organization (WHO) lists AMR among global health threats, with recent estimates, approximately 4.71 million deaths were associated with bacterial AMR in 2021, including 1.14 million deaths attributable to bacterial AMR. Based on the global analysis, more than 39 million deaths from antibiotic‐resistant infections are estimated to occur between 2025 and 2050 (Naghavi et al. 2024). These alarming figures reflect a crisis fueled by the overuse and misuse of antibiotics in health care, agriculture, and veterinary medicine, along with inadequate sanitation and infection control. Other risk factors for emergence of new pathogens and the spread of AMR are driven by globalization, increased mobility, population growth, frequent human–animal interactions, intensive agriculture, poor water and sanitation, environmental degradation, pollution, and climate change (Despotovic et al. 2023). Critically, AMR does not respect sectoral boundaries: drug residues and resistance genes from hospitals, farms, and communities accumulate in soil and water, enabling horizontal gene transfer between environmental bacteria and those colonizing humans or animals (Aladeeb 2025). Indeed, studies have highlighted that AMR gene pools in the environment can seed resistance in pathogens and that antibiotic use in one sector can drive resistance problems in another Perestrelo et al. (2023). This One Health paradigm which emphasizes the interdependence of human, animal, and ecosystem health is therefore essential to understanding and combating AMR.

Based on the list of 12 families of bacteria that pose the greatest threat to human health, the carbapenem‐resistant and extended‐spectrum β‐lactamases Enterobacteriaceae family is categorized as a critical priority for research and development of new antibiotics. Among the major AMR pathogens in Enterobacteriaceae family, *Escherichia coli* occupies a special place as a sentinel organism linking human, animal, and environmental reservoirs. So, the potential for bidirectional transfer of virulent or resistant *E. coli* (or their resistance genes) between animals and humans via direct contact, food, water, or fomites is the major concern (Poirel et al. 2018). *Escherichia coli* normally inhabits the gut of virtually all warm‐blooded animals, yet it can cause severe disease (e.g., urinary tract infections, sepsis and enteric infections) in humans and livestock (Alizade 2018), and it can acquire resistance genes from other bacteria and transfer them further (Poirel et al. 2018). The median change for the global level of resistance to imipenem in *E. coli* bloodstream infections (2018–2023) was 12.5% annually (95% CI: 9.4, 15.8), and resistance to cefotaxime increased in the Eastern Mediterranean Region by 5.8% (95% CI: 2.4, 9.6) (World Health Organization 2025).

Resistance to β‐lactam antibiotics in Gram‐negative bacteria such as *E. coli* is commonly associated with the production of β‐lactamase enzymes, including extended‐spectrum β‐lactamases (ESBLs) and carbapenemases (Bush and Bradford 2019). ESBLs hydrolyze the β‐lactam ring of Penicillins and third‐ and fourth‐generation cephalosporins and monobactams (Vrancianu et al. 2020). The largest group of ESBLs is the *bla*_*CTX‐M*_ type enzymes that are rapidly spreading among Enterobacteriaceae worldwide (Bevan et al. 2017; Rana et al. 2022). The *bla*_*CTX‐M*_ enzymes have spread extensively through mobile genetic elements such as transposons, integrons, and insertion sequences, particularly ISEcp1, which has played a key role in their global dissemination (Rana et al. 2022; D'Andrea et al. 2013). Some ESBLs and Carbapenemases have been associated with isolates that have high pandemic potential and spread across countries and continents (Livermore 2012).

Antimicrobial stewardship (AMS) programs in humans are highly effective strategies that optimize antibiotic use, minimize unnecessary prescriptions, improve clinical outcomes, and enhance safety (Abu‐Ajaleh et al. 2023). WHO has guided empirical antibiotic use via the Access, Watch, and Reserve (AWaRe) framework, emphasizing the risk of AMR from inappropriate antibiotic use. Access antibiotics are narrow‐spectrum, widely available, and recommended for first‐ and second‐line empirical treatments. Watch antibiotics are broader‐spectrum and considered priority targets for AMS because of their higher resistance potential, and they are more commonly used in hospitals. Severe hospital infections caused by multidrug‐resistant (MDR) organisms are recommended to be treated with reserve antibiotics (Zanichelli et al. 2023; Saleem et al. 2025). In veterinary medicine, AMS programs combine infection prevention and control with strategies that prioritize reducing antimicrobial use, reflecting the critical role of animals in transmitting AMR pathogens to humans (Lloyd and Page 2018). So, not only should a comprehensive framework include mandatory education for healthcare workers on responsible prescribing, but it should also implement formal stewardship programs across clinical settings and stricter regulatory oversight of antibiotic use in animal health and agriculture. Furthermore, national public awareness initiatives are vital to reducing inappropriate demand and fostering a collective understanding of antimicrobial resistance as a critical threat to society (World Health Organization 2025).

Environmental health, including water, sanitation, hygiene (WASH), waste management, and food safety, is essential to controlling AMR and reducing the spread of resistant organisms (Musoke et al. 2021). The constant overlap between human activities and livestock environments provides many opportunities for the transfer of antimicrobial‐resistant bacteria and the development or exchange of antimicrobial resistance genes (ARGs) (Bengtsson‐Palme et al. 2018; Helmy et al. 2023). Given the complexity of AMR, it is necessary to address it within the One Health framework (Velazquez‐Meza et al. 2022; Collignon and McEwen 2019).

Improved surveillance of antibiotic resistance in the One Health approach is essential for combating AMR (Haberecht et al. 2019). This approach enhances understanding of the complex interactions between human, animal, and environmental (Donkor et al. 2024) and ultimately leads to improved public health outcomes (Donkor et al. 2024; World Health Organization 2021).

Community‐acquired *E. coli* has become an increasingly important contributor to the global burden of AMR, with resistant strains now widely established beyond healthcare settings. Of particular concern is the rapid emergence and dissemination of MDR and ESBL‐producing *E. coli*, which are associated with limited therapeutic options and poorer clinical outcomes. A recent global systematic review and meta‐analysis estimated that 17.6% of healthy individuals carry intestinal ESBL‐producing *E. coli*, with community carriage increasing from 2.6% during 2001%–2005% to 26.4% during 2016–2020, reflecting the rapid global dissemination of these resistant strains. Marked geographical variation has been reported, with the highest prevalence observed in South‐East Asia (35.1%), followed by the Western Pacific (25.3%), Africa (21.4%), and the Eastern Mediterranean (20.6%), whereas substantially lower rates were reported in Europe (6.0%) and the Americas (10.3%). Consistent with these findings, systematic reviews from Asia have documented a high prevalence of MDR diarrheagenic *E. coli*, with high resistance to amoxicillin, ampicillin, nalidixic acid, tetracycline, and trimethoprim–sulfamethoxazole, while carbapenem resistance remains uncommon. Likewise, studies from Africa have identified widespread antimicrobial‐resistant *E. coli* in water sources, highlighting environmental reservoirs as important contributors to the persistence and dissemination of resistant bacteria and ARGs (Bezabih et al. 2022; Ramatla et al. 2023; Salleh et al. 2022). These observations emphasize that MDR and ESBL‐producing *E. coli* circulate across human, animal, and environmental sectors through interconnected transmission pathways. Accordingly, WHO adopted ESBL‐producing *E. coli* as the sentinel indicator organism in the Tricycle surveillance programme to support integrated AMR surveillance within a One Health framework and to monitor the emergence and dissemination of clinically important resistance at national, regional, and global levels (World Health Organization 2021).

In Iran, antimicrobial resistance in *E. coli* has increased substantially over the last decade. Evidence from systematic reviews and regional studies consistently demonstrates high resistance to commonly prescribed antibiotics, particularly ampicillin, trimethoprim–sulfamethoxazole, tetracyclines, and third‐generation cephalosporins, whereas carbapenems, amikacin, and nitrofurantoin remain among the most effective agents. Recent studies have also documented the increasing prevalence of ESBL‐producing *E. coli* and considerable geographical variation in resistance profiles across different provinces and healthcare settings, highlighting the growing challenge of empirical treatment and the need for continuous nationwide surveillance of antimicrobial resistance (Alizade 2018; Bahloli et al. 2024; Fakhri‐Demeshghieh et al. 2024).

Therefore, this study aimed to systematically assess the prevalence of antibiotic resistance and ARGs in *E. coli* isolates from human, animal, and environmental sources in Iran within a One Health framework by synthesizing published evidence from 2010 to January 2025. By integrating evidence across these interconnected sectors, this study seeks to provide a comprehensive overview of the distribution of resistance patterns and ARGs, identify existing knowledge gaps, and generate evidence to support national surveillance, antimicrobial stewardship, and evidence‐based policies for controlling antimicrobial resistance in Iran.

## 2 Methods

### 2.1 Search Strategy

The Scopus, Google Scholar, Magiran, PubMed, Scientific Information Database (SID), and Web of Science were searched. The library was searched for cross‐sectional studies on the prevalence of *E. coli* AMR isolates and ARGs in human, animal, food, and environment in Iran from 2010 to January 2025. Search terms included “*Escherichia coli*” OR “*E. coli*” AND “antibiotic resistance,” OR “drug resistance” OR “antimicrobial resistance” AND “Iran.” The study protocol was registered in PROSPERO with ID: CRD42023448322.

### 2.2 Study Selection

Initially, two review authors (FBA and AFD) screened the titles and abstracts of articles to identify the relevant studies. Subsequently, the full text of each eligible article was reviewed by at least two review authors independently (ZT/MA/AD). The FBA, AFD, and NB resolved the arising conflicts upon the selection of studies.

### 2.3 Inclusion Criteria

The present study included original research articles published in Persian or English that reported AMR of *E. coli* isolates from humans, animals, and environmental sources (including water and food) in Iran. Studies reporting AMR based on antibiotic susceptibility testing using the Minimum Inhibitory Concentration (MIC) method or the Disk Diffusion Method (DDM) were eligible for inclusion.

### 2.4 Exclusion Criteria

Studies were excluded if they met any of the following criteria: (i) some or all samples were collected before 2009; (ii) were review articles, systematic reviews, meta‐analyzes, or conference abstracts; (iii) did not follow recognized antimicrobial susceptibility testing guidelines, including the Clinical and Laboratory Standards Institute (CLSI) or the European Committee on Antimicrobial Susceptibility Testing (EUCAST); (iv) did not report precise AMR or ARG data as the primary or secondary outcomes; or (v) the year of sample collection could not be determined from the article (even after contacting the corresponding author). Based on a consensus among the study investigators, a±3‐year difference between the version of the CLSI guideline and the year of sample collection was considered acceptable.

### 2.5 Data Collection and Data Items

In this study, relevant data such as location, date of conducting and publishing the study, sample size, number of isolated *E. coli*, number of resistant isolates for each antibiotic, method of test, kind of sample, and study population (animals, humans, or environmental), and other relevant data were extracted from the articles and recorded in Microsoft Excel office software. Three review authors (Z.T.A., M.A., and A.D.) conducted the data extraction independently, and a fourth (F.B.A.) reviewed the information to ensure the validity. Any doubt about the study (except when there was no valid or clear CLSI), was tried to be solved by making contact with the authors. The year and period of sample collection, along with the source and type of isolates, were extracted from each included study when available. All antibiotics reported in the eligible studies were initially extracted. Antibiotics primarily targeting Gram‐positive bacteria were excluded from the analysis.

### 2.6 Quality Appraisal

The bias was primarily controlled by the use of the inclusion and exclusion criteria. At least two review authors (A.F.D., N.B., and A.D.) independently assessed the risk of bias by using the JBI Critical Appraisal Checklist for Studies Reporting Prevalence data (Munn et al. 2017). Conflicts that arose upon the quality appraisal were resolved by FBA. The appraisal was not used to assign weights or exclude studies based solely on an overall quality score. However, studies that did not meet the essential methodological requirements for valid and reliable identification and measurement of the outcome (JBI Questions 6 and 7) were excluded.

### 2.7 Analytic Approach

A random‐effect model was used for meta‐analysis using Stata software (version 17) for antimicrobial agents or ARGs if there were at least two study reports. If the individual studies reported proportions of 100 or 0 percent for an antimicrobial agent, a Freeman–Tukey double arcsine transformation was applied before the synthesis to stabilize the variance. The results were reported as pooled prevalence and 95% confidence intervals. Q and I^2^ statistics were used to assess statistical heterogeneity. In antibiotics with more than 10 studies, to assess the prevalence of drug resistance within a specified group and specific time‐points, random effects meta‐regression was used.

Data in this study were analyzed based on the One Health approach, including humans, animals, and the environment. In this study, food and environmental samples were reported as “environment” (Sagar et al. 2023).

For subgroup analysis, based on the time of study, the upper limit of the year of the study was considered. Time‐points were categorized based on important milestones of AMR in Iran:

– 2009–2015, the period before the establishment of the first National Action Plan of the Islamic Republic of Iran for combating antimicrobial resistance (NAP‐IRIAMR).
– 2016–2019, the period after the first NAP‐IRIAMR establishment and before the COVID‐19 pandemic.
– 2020–2025, the COVID pandemic years and after the pandemic.

In addition to calculating the pooled prevalence for each antibiotic for all isolates, the prevalence of resistance in classes of antibiotics (AB classes) (based on the classification CLSI 2025 M100‐Ed35 guideline) was calculated. The AMR in each class of antibiotics was built considering the maximum resistant isolates in each class. Subgroup analysis for antibiotics was considered for various categories, including pathotypes, time‐points, human groups, animal subgroups, and environment subgroups. The QGIS version 10.8 was used for mapping the AMR prevalence in antibiotic classes in different provinces of Iran. Changes of AMR during the time‐points were shown using GraphPad PRISMA software, Version 9.

To complement the review of extracted articles on genes, we identified and compiled sequence typing methods and corresponding allelic profiles reported in the studies.

## 3 Results

A total of 5194 studies were identified through the search of six databases, of which 368 were included in the meta‐analysis (Figure 1). AMR prevalence in *E. coli* isolates was assessed on 43,315 isolates. More than 90% of isolates (38,267 isolates, in 271 studies) belonged to the human samples, followed by animal (including 3851 isolates, in 51 studies), and environment (including 1945 isolates, in 38 studies) samples. The prevalence of detection of *E. coli from different populations* was 38.8 (95% CI: 36.5–41.2) (Table S1).

**Figure 1:**
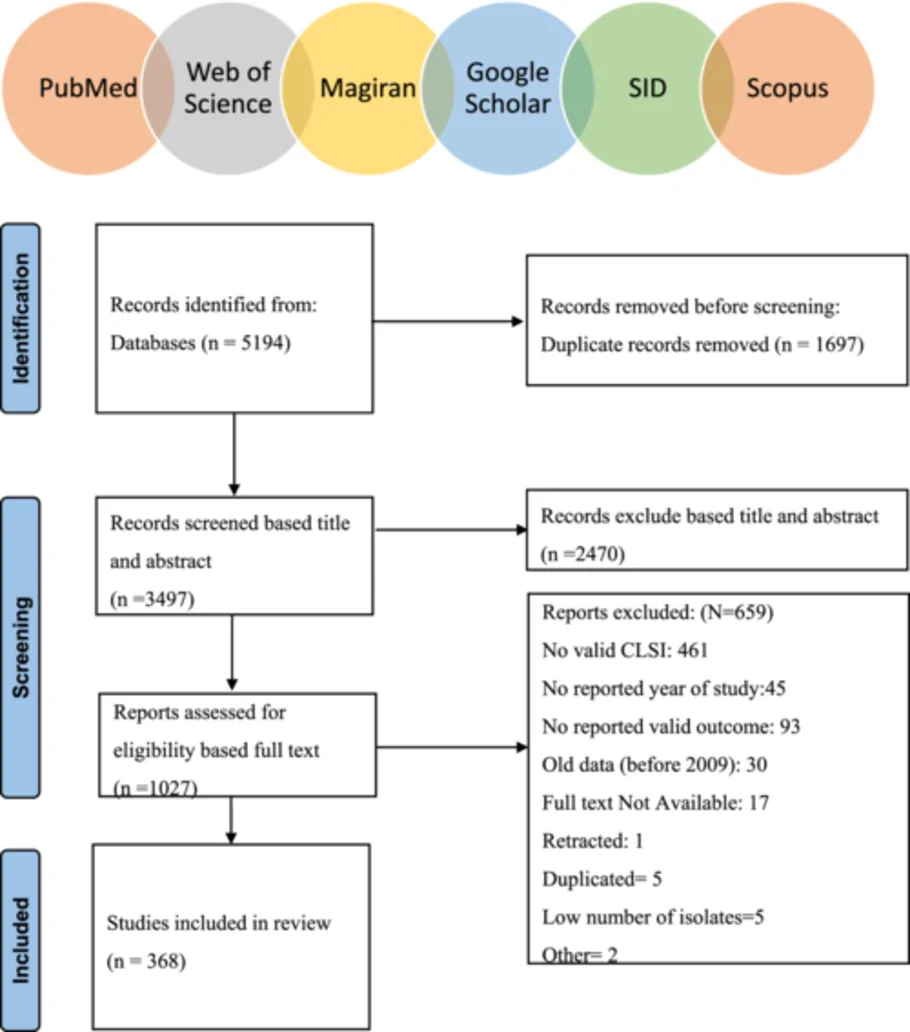
PRISMA flowchart, systematic review, and meta‐analysis for assessing antimicrobial resistance of Escherichia coli isolates from humans, animals, and environments in Iran from 2010 to 2025.

### 3.1 Pooled Prevalence in One Health Perspective *E. coli* Samples and Classes of Antibiotics

#### 3.1.1 AMR in Individual Antibiotics

Four antibiotics (Vancomycin, Oxacillin, Methicillin, Clindamycin) were excluded based on this criterion. All other antibiotics reported in the eligible studies were retained for analysis. Based on AWaRe classification (Lloyd and Page 2018), 18 antibiotics from the Access group, 37 from the Watch group, and 5 from the Reserve group were included in the meta‐analysis. Among antibiotics (with ≥ 5 number of primary studies), the highest resistance in Access, Watch, and Reserve was observed for Penicillin (82.7%, 95% CI: 77.3–88.2), Erythromycin (67.6%, 95% CI: 46.7–88.5), and Aztreonam (47.6%, 95% CI: 40.9–54.8), and the lowest resistance was observed for Nitrofurantoin (13.5%, 95% CI: 11.9–15.2), Doripenem (3.8%, 95% CI: 0.9–6.7), and Colistin (4.9%, 95% CI: 3.6–6.3), respectively (Table 1). The prevalence of AMR for Amikacin, Gentamicin, Norfloxacin, and Fosfomycin showed a decrease during time‐points, while some antibiotics like Ceftizoxime, Ertapenem, Streptomycin, Piperacillin‐Tazobactam, and Colistin surged with time (Figure S1).

**Table 1:** Prevalence of resistant isolates (%), 95% Confidence Interval (CI) among different groups of One Health isolates of *Escherichia coli* from Iran, and multivariable Meta‐regression for year and groups, during 2010–2025.

				Prevalence of AMR (95% CI)						Meta‐regression	
AWaRe	Antibiotics	Abbreviations	No of studies	Total	DEC	Environment	Healthy	Pathogenic	UPEC	Mean difference (95% CI)	
										Groups	Time‐point
**Access**	Amoxicillin/Clavulanic acid	AMC	88	50.0 (43.3–56.7)	48.8 (30.6–66.9)	67.5 (40.0–95.1)	35.2 (18.8–51.7)	55.7 (48.4–63.1)	38.4 (18.3–58.5)	0.2 (−2.8; 3.2)	−5.7 (−14.4; 2.9)
	Amikacin	AN	180	14.8 (13.6–16.0)	14.6 (10.7–18.5)	25.1 (17.2–32.9)	15.4 (9.2–21.5)	14.8 (13.3–16.3)	13.0 (9.9–16.1)	−0.3 (−0.9; 0.3)	**−2.3 (−3.9; −0.6)**
	Amoxicillin	AMX	58	71.2 (56.7–85.8)	81.7 (75.1–88.3)	70.0 (44.8–95.1)	63.3 (26.1–100.0)	69.5 (47.9–91.0)	84.1 (71.8–96.4)	−0.5 (−5.0; 4.1)	−7.8 (−2.5; 4.6)
	Ampicillin	AM	175	69.8 (66.5–73.2)	70.7 (62.1–79.4)	54.8 (35.0–74.7)	52.0 (35.7–68.3)	72.2 (67.6–76.7)	82.1 (77.0–87.1)	**2.5 (0.1; 4.9)**	−0.8 (−5.3; 3.6)
	Chloramphenicol	C	81	29.6 (24.1–35.0)	27.4 (20.2–34.7)	38.7 (20.2–57.1)	19.1 (11.1–27.2)	30.3 (23.0–37.6)	32.9 (0.0–67.7)	0.7 (−1.8; 3.2)	6.4 (−1.1; 13.9)
	Cephalothin	CF	43	55.7 (43.8–67.5)	41.0 (22.0–60.0)	40.4 (6.4–87.1)	46.6 (7.9–85.2)	65.9 (58.0–73.8)	56.6 (45.6–67.6)	7.0 (−0.9; 15.0)	−2.2 (−17.3; 12.9)
	Cephalexin	CN	31	56.7 (41.8–71.7)	74.2 (63.3–85.5)	42.2 (0.0–100.0)	20.3 (3.5–37.5)	63.1 (48.2–78.5)	59.3 (45.8–72.5)	4.9 (−2.0; 11.7)	1.9 (−15.0; 18.8)
	Cefazolin	CZ	51	62.2 (53.7–70.8)	71.7 (18.1–100.0)	—	10.5 (4.3–16.6)	68.5 (60.2–76.8)	59.3 (46.8–71.8)	3.6 (−2.1; 9.4)	1.5 (−9.6; 12.6)
	Doxycycline	DO	29	54.0 (38.8–69.1)	74.0 (48.8–99.3)	61.0 (21.2–100.0)	37.2 (3.0–71)	56.4 (33.1–79)	58.6 (0.0–100.0)	0.7 (−6.3; 7.7)	−2.1 (−19.3; 15.1)
	Nitrofurantoin	FM	131	13.3 (11.8–14.8)	10.8 (4.3–17.2)	41.9 (13.5–70.4)	8.3 (2.0–14.6)	12.3 (10.5–14.1)	12.7 (9.5–15.9)	**−1.3 (−2.3; −0.3)**	−2.1 (−15.0; 2.7)
	Gentamicin	GM	276	27.5 (25.0–30.1)	23.2 (16.8–29.7)	29.4 (17.9–41.0)	16.3 (7.7–25.0)	30.8 (27.4–34.2)	26.2 (21.6–30.9)	**1.5 (0.3; 2.7)**	**−9.9 (−13.2; −6.6)**
	Penicillin	P	18	82.7 (77.3–88.2)	85.2 (72.9–97.5)	57.7 (26.5–88.9)	91.9 (87.6–96.1)	90.2 (83.9–96.5)	100.0 (98.7–100.0)	**3.4 (0.6; 6.1)**	−1.7 (−10.0; 6.7)
	Ampicillin/Sulbactam	SAM	26	39.3 (28.4–50.3)	60.4 (0.0–100.0)	0.0 (0.0–2.5)	29.5 (2.3–56.6)	36.5 (21.1–51.8)	49.7 (35.2–64.2)	1.6 (−4.6; 7.8)	5.3 (−10.4; 21.1)
	Sulfamethoxazole	SMX	16	45.4 (27.6–63.2)	49.0 (3.8–94.3)	30.1 (0.0–72.3)	34.7 (0.0–86.0)	57.1 (23.8–90.4)	56.1 (44.4–67.7)	1.4 (−7.5; 10.3)	−15.5 (−16.0; 45.9)
	Trimethoprim‐ Sulfamethoxazole	TMP‐SMX	250	54.0 (49.8–58.2)	48.6 (35.3–62.0)	41.5 (28.4–54.6)	32.1 (24.0–40.3)	59.7 (56.0–63.5)	60.5 (55.9–65.1)	**3.2 (1.3; 5.2)**	2.2 (−3.3; 7.8)
	Tetracycline	TE	153	59.1 (52.4–65.8)	63.6 (56.9–70.2)	60.7 (36.7–84.7)	43.2 (33.8–52.7)	61.1 (48.4–73.8)	62.1 (55.9–68.3)	0.2 (−2.7; 3.1)	−2.4 (−11.5; 6.6)
	Sulfadiazine/trimethoprim	TMP/SDZ	6	67.0 (57.3–76.7)	37.5 (4.0–71.0)	80.0 (44.9–100.0)	—	68.2 (58.0–78.4)	—	—	—
	Trimethoprim	TMP	23	48.1 (36.5–59.7)	45.6 (27.5–63.7)	20.5 (3.1–38.0)	70.4 (56.9–83.9)	49.5 (17.8–81.2)	51.7 (36.7–66.8)	1.9 (−3.5; 7.3)	−4.1 (−19.1; 10.8)
**Watch**	Azithromycin	AZM	23	39.8 (26.5–53.2)	44.3 (23.9–64.7)	76.5 (70.6–82.4)	44.9 (14.0–75.8)	47.5 (39.3–55.7)	53.4 (40.9–65.9)	0.6 (−2.6; 3.9)	3.5 (−6.1; 13.2)
	Carbenicillin	CB	3	34.4 (0.0–75.9)	—	0.0 (0.0–2.0)	—	51.9 (32.4–71.4)	—	—	—
	Ceftazidime	CAZ	210	43.6 (39.9–47.4)	33.3 (23.6–42.9)	44.4 (26.6–62.3)	32.3 (22.4–42.2)	46.9 (41.8–51.9)	48.9 (42.1–55.6)	**2.7 (0.87; 4.4)**	0.9 (−4.0; 5.9)
	Cefixime	CFM	71	45.1 (39.5–50.7)	43.9 (29.5–58.4)	23.7 (12.2–35.1)	29.8 (10.2–49.4)	48.0 (40.8–55.2)	46.9 (36.2–57.6)	1.9 (−0.8; 4.6)	−2.0 (−9.9; 5.9)
	Ciprofloxacin	CIP	283	41.4 (38.1–44.7)	23.6 (18.8–28.3)	47.4 (30.4–64.4)	33.8 (22.7–44.8)	45.5 (41.0–49.9)	47.2 (41.5–53.0)	**3.2 (1.8; 4.6)**	2.8 (−1.2; 6.8)
	Clarithromycin	CLR	2	32.5 (0.0–69.1)	12.5 (0.0–35.4)	50.0 (36.7–63.3)	—	—	—	—	—
	Cefpodoxime	CPD	19	66.4 (45.1–87.7)	71.0 (37.4–100.0)	—	63.3 (51.1–75.5)	63.5 (33.0–94.1)	70.7 (57.1–84.2)	−5.8 (−10.7; 9.5)	4.4 (−21.1; 29.8)
	Cefoperazone	CPR	3	47.3 (0.0–100.0)	—	—	0.0 (0.0–13.5)	70.0 (12.9–100.0)	—	—	—
	Ceftriaxone	CRO	155	46.6 (41.2–52.1)	32.5 (23.6–41.5)	40.0 (16.5–63.6)	27.4 (14.7–40.0)	51.9 (43.9–59.8)	53.3 (39.3–67.3)	**3.8 (1.2; 6.3)**	3.2 (−4.1; 10.4)
	Cefuroxime	CXM	18	57.2 (38.1–76.4)	29.1 (0.0–86.9)	—	66.7 (13.3–100.0)	61.2 (37.7–84.8)	55.6 (40.2–100.0)	7.2 (−4.6; 18.9)	−9.1 (−36.1; 18.0)
	Chlortetracycline	CTC	3	65.3 (0.0–100.0)	—	—	—	65.3 (0.0–100.0)	—	—	—
	Cefotaxime	CTX	213	49.1 (44.5–53.8)	38.3 (29.9–46.6)	31.7 (16.0–47.3)	37.7 (24.1–51.2)	53.6 (47.3–59.9)	57.8 (51.1–64.5)	**3.6 (1.5; 5.6)**	3.5 (−2.3; 9.3)
	Ceftizoxime	ZOX	21	34.6 (25.7–43.5)	48.5 (11.8–85.2)	—	21.6 (0.0–46.2)	32.7 (23.1–42.3)	40.5 (16.4–64.7)	0.7 (−3.4; 4.8)	**16.4 (1.6; 31.2)**
	Doripenem	DOR	6	3.8 (0.9–6.7)	—	—	0.0 (0.0–2.3)	6.8 (2.3–11.3)	0.0 (0.0–2.3)	—	—
	Erythromycin	E	31	67.6 (46.7–88.5)	83.7 (74.5–93.0)	45.9 (5.8–85.9)	56.8 (15.4–98.2)	72.7 (37.7–100.0)	60.0 (43.8–76.2)	1.5 (−7.5; 7.8)	6.1 (−13.8; 26.1)
	Enrofloxacin	ENR	31	42.5 (27.0–57.9)	30.5 (15.6–45.4)	51.7 (12.3–91.1)	34.0 (11.7–56.3)	54.7 (21.2–88.1)	—	—	—
	Ertapenem	ETP	24	4.7 (3.3–6.1)	0.6 (0.0–1.7)	—	0.0 (0.0–2.3)	7.4 (5.3–9.4)	0.5 (0.0–2.1)	0.6 (−0.5; 1.8)	**5.5 (3.1; 7.9)**
	Cefepime	FEP	92	40.6 (33.8–47.3)	35.2 (14.9–55.4)	21.3 (0.0–53.8)	34.3 (11.6–57.0)	42.8 (36.1–49.4)	44.9 (21.3–68.5)	2.8 (−0.2; 5.8)	−2.7 (−9.6; 4.1)
	Cefoxitin	FOX	63	30.8 (23.9–37.6)	29.4 (7.9–50.8)	20.8 (8.2–33.4)	22.8 (8.9–36.7)	36.8 (28.3–45.3)	15.8 (8.5–23.1)	0.6 (−2.1; 3.3)	−0.7 (−8.7; 7.3)
	Flumequine	FLU	8	62.3 (19.1–100.0)	44.2 (5.5–83.0)	—	70.9 (52.4–89.3)	68.3 (4.5–100.0)	—	—	—
	Gatifloxacin	GAT	3	59.9 (53.9–65.8)	—	—	—	59.9 (53.9–65.8)	—	—	—
	Kanamycin	K	29	22.9 (17.3–28.5)	16.3 (8.1–24.5)	59.2 (7.2–100.0)	13.8 (8.3–100.0)	28.6 (13.7–100.0)	22.7 (17.4–28.0)	−0.3 (−3.1; 2.4)	3.5 (−4.5; 11.5)
	Lincomycin	LIN	4	62.5 (8.8–100.0)	24.5 (8.2–40.8)	100.0 (77.9–100.0)	—	100.0 (97.1–100.0)	—	—	—
	Lomefloxacin	LOM	2	83.0 (57.1–100.0)	—	100.0 (67.6–100.0)	72.7 (54.1–91.3)	—	—	—	—
	Levofloxacin	LVX	45	34.5 (27.4–41.5)	18.1 (6.5–29.6)	11.4 (0.0–26.5)	10.7 (4.5–16.8)	40.6 (30.8–50.3)	43.9 (29.5–58.3)	**8.2 (2.3; 14.1)**	−0.8 (−9.7; 8.1)
	Meropenem	MEM	81	7.3 (5.0–9.5)	0.7 (0.0–1.5)	0.0 (0.0–2.9)	1.9 (0.0–4.2)	9.0 (5.9–12.1)	3.7 (1.5–5.9)	1.1 (−0.1; 2.4)	3.0 (−2.9; 6.0)
	Mezlocillin	MEZ	3	40.7 (4.5–76.7)	—	—	20.0 (0.0–44.8)	50.2 (8.5–91.9)	—	—	—
	Minocycline	MI	3	29.0 (21.9–36.1)	30.0 (13.6–46.4)	—	—	28.8 (19.8–37.7)	—	—	—
	Neomycin	NEO	15	38.3 (12.4–64.3)	23.5 (18.1–28.9)	100.0 (77.9–100.0)	13.4 (8.7–13)	41.2 (0.0–41)	56.5 (45.9–67.0)	3.5 (−9.8; 16.7)	8.2 (−27.4; 43.8)
	Netilmicin	NET	2	26.3 (22.3–30.3)	—	—	—	26.3 (22.3–30.3)	—	—	—
	Norfloxacin	NX	52	37.1 (32.6–41.5)	11.1 (0.0–31.6)	51.7 (12.5–91.0)	14.6 (6.2–22.9)	37.3 (30.4–44.2)	47.2 (40.6–53.8)	2.9 (−0.2; 6.0)	**−8.2 (−15.9; −5.5)**
	Ofloxacin	OFL	37	39.9 (34.2–45.2)	21.9 (0.0–55.2)	25.9 (14.2–37.6)	20.0 (0.0–44.8)	42.0 (35.2–48.8)	43.0 (34.7–51.3)	**5.6 (0.5; 10.7)**	2.2 (−6.2; 10.7)
	Oxytetracycline	OTC	15	50.1 (31.3–69.0)	68.0 (59.3–76.8)	40.0 (18.5–61.5)	47.2 (26.3–68.0)	51.2 (14.6–87.9)	—	0.8 (−11.1; 12.8)	10.4 (−32.7; 53.6)
	Piperacillin	PIP	37	47.0 (35.3–58.8)	50.5 (7.3–93.6)	49.7 (17.7–81.7)	1.5 (0.0–6.2)	51.2 (34.1–68.4)	44.3 (26.0–62.6)	2.2 (−4.1; 8.5)	3.9 (−12.2; 19.9)
	Streptomycin	STR	43	14.2 (2.9–25.5)	38.0 (29.4–46.6)	36.5 (0.0–80.9)	23.7 (15.2–32.1)	32.3 (18.7–45.9)	39.3 (13.6–65.0)	−0.8 (−5.1; 3.4)	**14.2 (2.9; 25.5)**
	Tobramycin	TM	37	33.5 (26.2–40.8)	26.9 (4.6–49.2)	0.0 (0.0–2.9)	55.7 (20.5–91.0)	35.2 (25.1–45.3)	7.2 (0.0–17.2)	4.6 (−4.2; 5.1)	−8.9 (−19.7; 1.9)
Piperacillin‐ Tazobactam	TZP	51	17.3 (14.5–20.0)	15.4 (0.0–39.2)	—	34.1 (6.9–61.2)	16.5 (13.3–19.8)	16.8 (11.6–22.1)	–0.2 (−3.6; 3.3)	**4.2 (0.2; 8.2)**
**Reserve**	Aztreonam	ATM	71	47.9 (40.9–54.8)	44.3 (23.9–64.7)	76.5 (70.6–82.4)	44.9 (14.0–75.8)	47.5 (39.3–55.7)	53.4 (40.9–65.9)	0.6 (−2.6; 3.9)	3.5 (−6.1; 13.2)
	Colistin	CL	27	4.9 (3.6–6.3)	8.1 (3.5–12.7)	46.3 (0.0–100.0)	18.5 (0.0–41.6)	3.9 (2.5–5.3)	—	−1.1 (−2.9; 0.6)	**2.2 (0.2; 4.3)**
	Tigecycline	TGC	6	15.4 (8.9–21.9)	—	—	—	15.4 (8.9–21.9)	—	—	—
	Fosfomycin	FOS	27	8.0 (5.9–10.1)	6.5 (0.0–13.3)	—	1.0 (0.0–2.8)	11.2 (8.0–14.4)	4.8 (1.4–8.3) + H52	0.6 (−0.9; 2.1)	**−5.3 (−8.7; −2.1)**
	Polymyxin‐B	PB	2	10.5 (0.0–33.7)	25.0 (3.8–46.2)	—	—	0.9 (0.0–2.6)	—	—	—
**Not in AWaRe**	Imipenem	IMP	217	11.9 (10.4–13.3)	8.1 (3.7–12.4)	21.3 (14.2–28.4)	4.7 (2.4–7.0)	13.5 (11.4–15.6)	6.0 (4.2–7.8)	0.4 (−0.4; 1.1)	−2.0 (−0.3; 3.8)
	Nalidixic acid	NA	146	55.8 (50.4–61.2)	48.4 (28.4–68.4)	45.4 (12.7–78.1)	38.5 (15.3–61.7)	59.2 (53.2–65.1)	64.0 (58.0–70.0)	**5.1 (0.7; 9.4)**	0.3 (−7.2; 7.8)
	Ceftiofur	CFT	2	38.7 (0.0–100.0)	—	—	—	0.0 (0.0–1.2)	77.6 (68.8–86.5)	—	—
	Danofloxacin	DAN	5	40.1 (1.1–79.0)	11.1 (0.0–31.6)	—	—	47.0 (2.2–91.8)	—	—	—
	Lincospectin	LS	10	8.1 (4.6–11.6)	0.0 (0.0–1.4)	—	2.3 (0.0–6.8)	19.3 (8.4–30.3)	2.4 (0.0–5.6)	3.8 (−1.8; 9.4)	−0.6 (−8.8; 7.5)
	Furazolidone	AOZ	4	27.3 (6.9–47.7)	—	34.9 (16.6–53.2)	8.0 (0.5–15.5)	—	—	—	—
	Florphenicol	FLO	14	28.2 (17.9–28.5)	23.1 (0.0–58.6)	—	18.9 (6.1–31.8)	38.0 (6.9–69.1)	—	6.8 (−6.8; 20.4)	8.1 (−12.8; 28.7)
	Ticarcillin‐clavulanate	TIM	2	66.2 (57.0–75.4)	71.3 (63.9–78.9)	—	—	62.0 (57.6–66.4)	—	—	—

#### 3.1.2 AMR in Classes of Antibiotics

Antibiotics in this study belonged to 16 classes of antibiotics based on CLSI 2025. The highest resistance was observed in the classes of Penicillins (71.7%, 95% CI: 65.6–76.6), Macrolides (58.9%, 95% CI: 46.9–71.0), Tetracyclines (58.9%, 95% CI: 52–65.9), and Cephems (57.3%, 95% CI: 53.6–61.1). In contrast, the lowest AMR was seen in classes of Fosfomycin (6.8%, 95% CI: 5.2–8.5), Lipopeptides (10.5%, 95% CI: 8.4–12.6), and Penems (12.8%, 95% CI: 11–14.6), and Nitroheterocyclics (13.5%, 95% CI: 12.0–15.0) (Tables S3 and S4).

The pattern of AMR across different classes differed between humans, animals, and the environment; the class of Penicillins in humans and the environment, and the class of Macrolides in animals, showed the highest resistance. However, the class of Fosfomycins in humans, and Penems in both animals and environment showed the lowest resistance (Figures 2 and S2–S4).

**Figure 2:**
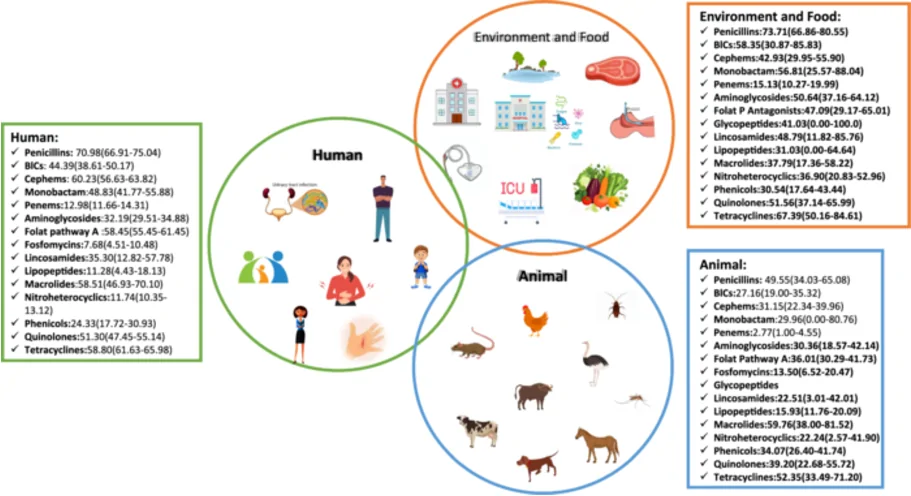
Prevalence of resistance in Escherichia coli isolates in classes of antibiotics in terms of humans, animals, and environments.

Neither province had AMR prevalence higher than 40% in the classes of Penems, Fosfomycins, and Nitroheterocyclics (Figure 3 and Table S2). Also, different classes showed different patterns of AMR in human (Figure S2), animal (Figure S3), and environment (Figure S4), but in the One Health perspective, the prevalence of classes of Aminoglycosides and Fosfomycins showed a decreasing trend during time‐points (Table S3).

**Figure 3:**
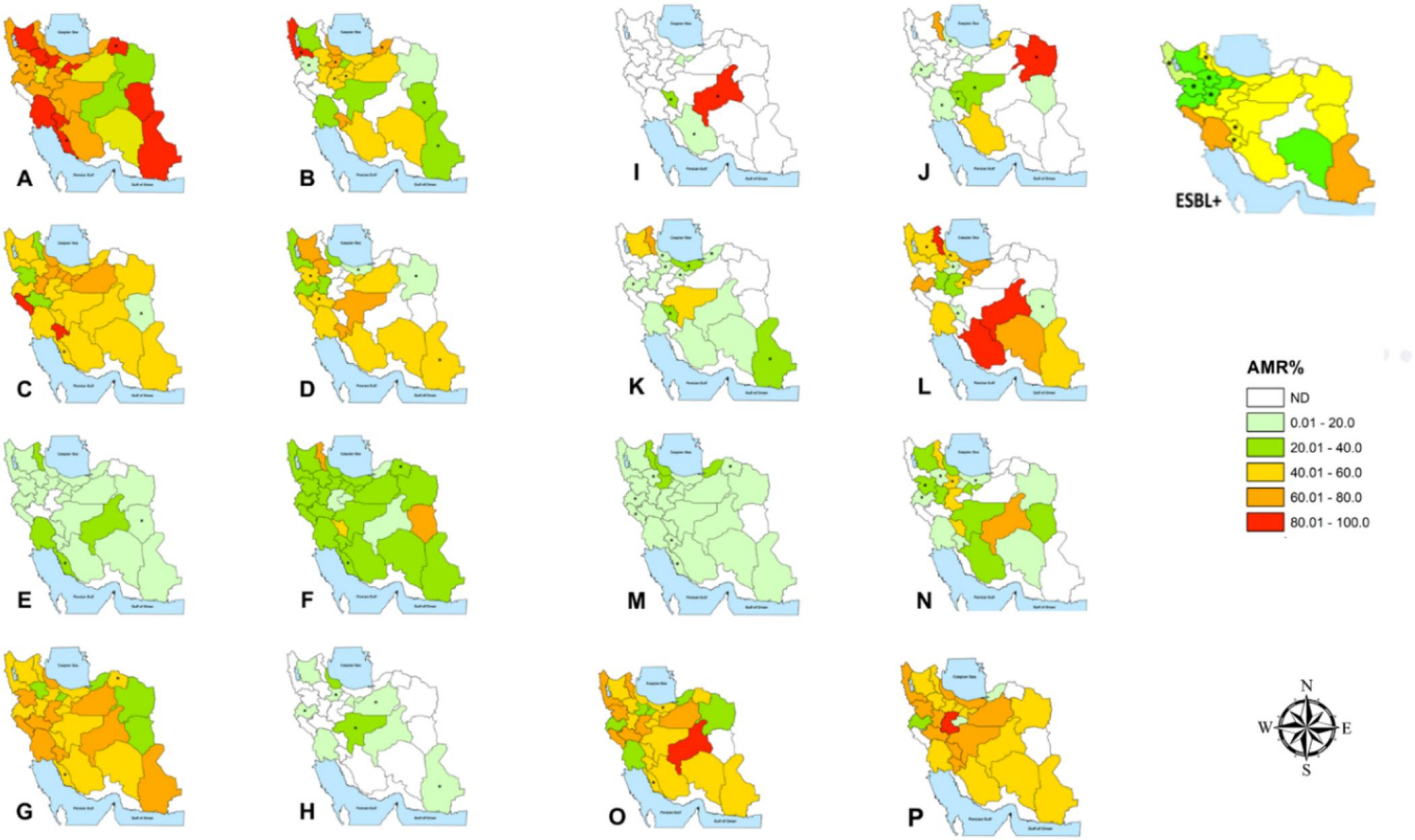
Spatial distribution of AMR of Escherichia coli in different classes of antibiotics (A) Penicillins, (B) β‐lactam combination agents, (C) Cephems, (D) Monobactams, (E) Penems, (F) Aminoglycoside, (G) folate pathway antagonists, (H) Fosfomycin, (I) Glycopeptides, (J) Lincosamides, (K) lipopeptides, (L) Macrolides, (M) Nitroheterocyclic, (N) Phenicols (O) quinolones, (P) Tetracycline. The Prevalence of ESBL‐producing *Escherichia coli* is shown in the ESBL+ map. The * indicates the result of a single study, and the prevalence is not related to the pooled prevalence.

### 3.2 Pooled Prevalence in Human Samples

Totally, 271 Studies with 38267 isolates reported the prevalence of AMR in human samples. The highest resistance in human samples was for Penicillin (99.6%), Erythromycin (89.1%), and Amoxicillin (75.5%); hence, the lowest resistance had belonged to Doripenem (3.8%) and Ertapenem (4.9%) (Table S4). Resistance to Ciprofloxacin showed a statistically significant increase over the time points, as well as Cefotaxime, Ertapenem, Kanamycin, and Imipenem. In contrast, Erythromycin and Gentamicin showed decreases over time (Figure S5).

### 3.3 Pooled Prevalence in Animal Samples

Generally, 51 studies with 3851 isolates were found for animal samples. The highest prevalence of resistance was seen for Flumequine (62.3%) and Erythromycin (61.1%), but Meropenem (1.8%) and Imipenem (2.8%) showed the lowest resistance (Table S5). Meta‐regression analysis revealed a significant increase in resistance to Chloramphenicol, Trimethoprim‐Sulfamethoxazole, Streptomycin, and Florfenicol during the time‐points. Conversely, significant decreases were observed for Amikacin and Gentamicin (Figure S6).

### 3.4 Pooled Prevalence in Environment Samples

Totally, 38 studies with 1945 isolates reported the prevalence of AMR in environmental samples (including food and environment). The overall prevalence of AMR varied considerably among antibiotics, with the highest resistance rates observed for Amoxicillin (71.8%), Tetracycline (67.3%), and Ampicillin (63.7%). The *E. coli* isolates showed the lowest resistance for Imipenem (15.4%) and Cefoxitin (16.2%) (Table S6). Meta‐regression analysis revealed a significant decrease in AMR during the time‐point period for Amikacin, Ampicillin, and Gentamicin. Conversely, Streptomycin showed a statistically significant increase in AMR during the same period (Figure S7).

### 3.5 ESBL‐Producing *E. Coli*

#### 3.5.1 Prevalence of ESBL Isolates

Overall, 7,885 out of 18,323 *E. coli* isolates (47.2%, 95% CI: 39.6–54.7) were phenotypically classified as ESBL‐producing (in 136 studies). A total of 7637 of 17,385 isolates (46.2%, 95%: 40.2–53.0) in human samples, 49 of 370 (11.8%, 95% CI: 3.5–20.2) of animal samples, and 77 of 373 isolates (42.0%, 95% CI: 9.9–74.2) of environmental samples (including food and environment) were reported as ESBL‐producing *E. coli* (Table S1). Also, the highest prevalence of ESBL‐producing isolates was seen in Sistan and Baluchistan (65.3%) and Khuzestan (64.9%) (Figure 3 and Table S7).

### 3.6 Pooled Prevalence of AMR in ESBL‐Producing *E. Coli*

This meta‐analysis included 5,804 isolates from 70 studies that phenotypically confirmed ESBL status (ESBL‐positive: 3510 isolates, and ESBL‐negative: 1898 isolates). The population was predominantly human (69 studies), with one animal study. Pooled prevalence estimates demonstrated significantly higher resistance rates in ESBL‐producing isolates compared to ESBL‐negative isolates for all antibiotics except fosfomycin. Meta‐regression analysis confirmed this significant association for a range of both β‐Lactams (Cephalexin, Cefazolin, Ceftazidime, Cefixime, Ceftriaxone, Cefotaxime, Cefepime, and Imipenem), and Non‐β‐Lactams (Ciprofloxacin, Levofloxacin, Nalidixic Acid, Gentamicin, and Trimethoprim‐Sulfamethoxazole. antibiotics) (Table S8).

### 3.7 Pooled Prevalence of β‐lactamase ARGs and Co‐Occurrence in ESBL–Producing Isolates

The pooled prevalence of ARGs was determined from 3,714 isolates. The most prevalent gene was *bla*_CTX‐M_ (68.2%) followed by *bla*_TEM_ (59.4%), *bla*_SHV_ (24.2%), and *bla*_OXA_ (20.1%) (Table S9). Gene co‐occurrence analysis revealed that the combined presence of *bla*_CTX‐M_ and *bla*_TEM_ was the most frequent (23.3%). The pooled prevalence for other combinations was lower: *bla*_CTX‐M/_ *bla*_SHV_ at 7.6%, and *bla*_TEM*/*_ *bla*_SHV_ at 8.3%. The co‐occurrence of all three genes (*bla*_CTX‐M,_ *bla*_TEM_, and *bla*_SHV_) was observed in 9.2% of isolates.

### 3.8 AmpC‐Producing Isolates

#### 3.8.1 Prevalence of AmpC‐Producing Isolates

Based on phenotypic assessment of 2677 *E. coli* isolates from 21 studies, 397 isolates were defined as AmpC (20.2%, 95% CI: 15.0–25.4). All the samples belonged to humans, except one study, so the prevalence in humans was 21.7% (95% CI: 16.4–‐27.1) (Table S1).

### 3.9 Prevalence of β‐lactamase ARGs in AmpC‐Producing Isolates

A total of 198 AmpC isolates were assessed using a molecular assay for the characterization of ARGs. The ascending prevalence of ARGs in AmpC isolates was *bla*_*CIT*_ (62.2%), *bla*_*CMY*_ (44.9%), *bla*_*ACC*_ (30.5%), *bla*_*DHA*_ (18.9%), *bla*_*EBC*_ (10.8%), *bla*_*FOX*_ (6.2%), *bla*_*NDM*_ (4.1%), and *bla*_*MOX*_ (1.7%), respectively (Table S9).

### 3.10 Metallo‐β‐Lactamase (MBL)‐Producing Isolates

#### 3.10.1 Prevalence of MBL‐Producing Isolates

The pooled prevalence of MBL production among 2368 *E. coli* isolates from 11 studies was 14.1% (95% CI: 9.5–18.7), corresponding to 155 confirmed MBL‐producing isolates. All analyzed isolates originated from human samples (Table S1).

### 3.11 Prevalence of β‐lactamase ARGs in MBL‐Producing Isolates

The ARGs were assessed in 102 MBL isolates of five studies. The most frequent ARGs were *bla*_*NDM*_ (30.1%), *bla*_*IMP*_ (22.8%), and *bla*_*OXA‐48*_ (5.8%) (Table S9).

We extracted the typing methods and sequence types reported in the studies. Notably, there was little use of the MLST framework among the extracted studies, while other methods were more prevalent. The financial and operational challenges of the MLST method appear to be factors influencing this choice (Table S10).

## 4 Discussion

This systematic review and meta‐analysis provides a comprehensive One Health assessment of AMR in Escherichia coli across human, animal, and environmental reservoirs in Iran. Across 368 studies, resistance was widespread, although the magnitude and pattern of resistance varied substantially by antimicrobial class and reservoir. High resistance was observed for several commonly used antimicrobial classes, while ESBL, AmpC, MBL production and clinically important resistance genes were also frequently reported. Importantly, the temporal analyzes identified significant increases in resistance to selected antimicrobials, indicating that resistance patterns are not static and require continued surveillance. The high resistance estimates observed in Iran are broadly consistent with the high burden of AMR reported from other low‐ and middle‐income settings, particularly for commonly used β‐lactams (Naghavi et al. 2024). However, direct comparison across countries should be made cautiously because resistance estimates are influenced by differences in population characteristics, specimen types, healthcare settings, antimicrobial susceptibility testing methods, and surveillance coverage.

The sector‐specific resistance profiles observed in this review suggest that antimicrobial selection pressures differ across One Health reservoirs. Human isolates showed high resistance to several frequently prescribed clinical antimicrobials, whereas animal isolates demonstrated distinct resistance patterns involving antimicrobials commonly used in veterinary and food‐animal settings. Environmental isolates showed particularly high resistance to several older and widely used agents, consistent with the potential role of wastewater, agricultural runoff, and other contaminated environments in maintaining and disseminating resistant organisms. Nevertheless, these findings should not be interpreted as evidence of direct transmission between sectors because the included studies did not provide sufficient longitudinal or genomic data to establish transmission pathways. But critically, the distinct resistance profiles observed across human, animal, and environmental compartments underscore that AMR in Iran, as elsewhere, is an inherently intersectoral phenomenon that cannot be effectively addressed outside a One Health framework (Velazquez‐Meza et al. 2022; Collignon and McEwen 2019).

The increasing prevalence of AMR and ARGs has become a global problem, resulting in increased economic costs, more difficult‐to‐treat infections, and mortality (Chinemerem Nwobodo et al. 2022). This systematic review and meta‐analysis provides a comprehensive One Health assessment of AMR in *Escherichia coli*, integrating phenotypic resistance patterns, ESBL/AmpC/MBL production, and ARGs across human, animal, and environmental compartments in Iran. The results demonstrate that AMR in *E. coli* is widespread across all One Health sectors, but with distinct resistance profiles reflecting sector‐specific antibiotic pressures. Our data from 2010 to 2025 demonstrates alarmingly high resistance to Penicillins, Tetracyclines, Macrolides, Cephems and Quinolones based on all isolates, while Fosfomycins, Lipopeptides, and Penems showed low resistance.

Based on the WHO AWaRe classification, our findings highlighted concerning resistance patterns across Access, Watch, and Reserve antibiotics in the One Health approach. Within the Access group, high resistance was observed for common first‐line agents such as Penicillin and Ampicillin, which raises major concerns given their essential role in empirical therapy. Among the Watch group, Erythromycin and Cefpodoxime showed alarmingly high resistance. Aztreonam and Tigecycline as Reserve antibiotics, which should be preserved for treatment of MDR pathogens, were seen as alarming resistance. Based the ICDC reports, only 48.6% of antibiotics consumptions of Iranian people in 2024 belonged to Access groups and 51.3% were related to Watch groups (ICDC 2025) while according to AWaRew Antibiotic Book, at least 60% of antibiotics should be from Access groups (World Health Organization 2022), and this gap shows the requirement of more supervision. Fortunately, some antibiotics like Nitrofurantoin and Amikacin from Access group; Doripenem and Meropenem from Watch group; and Colistin and Fosfomycin still show low resistance, and are largely effective. AMS programs are defined as a set of coordinated strategies that promote responsible antimicrobial use and should be designed within a One Health framework, simultaneously addressing human, animal, and environmental health (Hibbard et al. 2024; Shrestha et al. 2023). Although the trend of AMR in One Health view, for some antibiotics was decreasing, but Ceftizoxim, Ertapenem, Streptomycin, Piperacillin‐Tazobactam from the Watch group, and Colistin from the Reserve showed an increasing trend over time. As a result of other studies, unnecessary antibiotic use was observed during COVID‐19 (Petrakis et al. 2023), and prescribing increased by 87% in 2020–2021 compared to 2019–2020. This ratio increased even more (94%) in 2021–2022. This surge was driven by the empirical use of Azithromycin (for respiratory symptoms) and β‐lactams (e.g., Amoxicillin, Cephalexin) (Alijaniha et al. 2025), and may have contributed to antimicrobial selection pressure. A recent study reported a decline in the Access‐to‐Watch Index from 0.96 in 2016 to 0.92 in 2020, indicating high consumption of Watch antibiotics in LMICs (Saleem et al. 2025). Watch and Reserve groups warrant careful prescribing because of their increased resistance potential. In addition to the toxicity of the Watch and Reserve antibiotics in some cases at higher doses, they are considered more advanced antibiotics with a higher potential for resistance in comparison to the Access group, and subsequently higher costs, more difficult treatment, higher mortality, and encountering more severe resistance in a shorter period of time (Mugada et al. 2021; Zanichelli et al. 2023). However, resistance prevalence alone cannot determine the appropriateness of individual prescribing practices. Therefore, AWaRe‐based interpretation should be integrated with national antimicrobial consumption data, clinical indications, and local treatment guidelines.

Highest resistance in human samples was seen for Penicillin, Erythromycin, and Amoxicillin, hence, Doripenem and Ertapenem still had remained in low resistance. Resistance to some antibiotics of Quinolones (Ciprofloxacin), Cephems (Cefotaxime), Penems (Ertapenem and Imipenem), and Aminoglycosides (Kanamycin,) showed linear increase during time. Based on the national surveillance report, Macrolids (33.8%), Penicilinse (22.1%), Cephems (Third‐generation; 16.1%) and Quinolones (10%) consisted the most used antibiotic in Iran in 2024, while only 0.2% of antibiotics belonged to Penems (ICDC 2025). Despite low resistance against Imipenem (11%), the trend was increasing. This upward trajectory is consistent with global reports from the WHO GLASS, which documented a significant increase in imipenem resistance to 12.5% (95% CI: 9.4–15.8) (World Health Organization 2025). This trend is reflected in recent national surveillance data from Iran, which has reported a substantially higher resistance rate of 20% among both inpatient and outpatient *E. coli* isolates (ICDC 2025). As a carbapenem, imipenem is a last‐line antibiotic crucial for treating severe infections caused by Gram‐negative bacteria (Larsson and Flach 2022). Therefore, this significant and consistent increase in resistance observed both globally and more acutely at the national level represents a critical public health warning. The convergence of these trends, particularly the high baseline rate documented in Iran, underscores the urgent need for rigorous antimicrobial stewardship programs. Without the implementation of comprehensive monitoring and strict control over antibiotic usage, the continued escalation of imipenem resistance will likely render these infections untreatable, leading to a major health crisis in the near future.

ESBLs, class A Ambler and Bush–Jacoby, are enzymes that hinder the effectiveness of β‐lactam antibiotics, including Penicillins, 3rd and 4th generation Cephalosporins, and Monobactams (Shrestha et al. 2023; Petrakis et al. 2023; Alijaniha et al. 2025). A central finding of this analysis is the high prevalence of extended‐spectrum β‐lactamase (ESBL) production, which was detected in nearly half (46.2%) of all E. coli isolates studied. This high rate was driven primarily by human isolates (48.2%), but notably, environmental samples also showed a substantial prevalence (42.0%), indicating a widespread circulation of these resistant strains beyond clinical settings. In our study, in addition to the Cefixime, Ceftazidime, Cefotaxime, and Cefepime, the prevalence of AMR for Cephalexin, Cefazolin, Ceftriaxone, Ciprofloxacin, Imipenem, Gentamicin, Trimethoprim‐Sulfamethoxazole, Levofloxacin, and Nalidixic acid in ESBL‐producing isolates was significantly higher in ESBL‐producing isolates in comparison to non‐ESBL isolates. In a study conducted on hospitalized patients, 71.2% (Salleh et al. 2022), and in another study on suspected COVID‐19 patients, 75.9% of all *E. coli* were ESBL‐producing (Mugada et al. 2021). Based on the result of a systematic review and meta‐analysis study, during 2008 and 2018, 43.2% of isolates were phenotypically ESBL‐producing (Zanichelli et al. 2023).

Among all preferred or alternative antibiotics in IDSA 2024 Guidance (Infectious Diseases Society of America) for uncomplicated cystitis, Pyelonephritis or cUTIs, and Infections outside of the urinary tract induced by ESBL‐producing Enterobacterales (ESBL‐E), the Carbapenems, Fosfomycin and Nitrofurantoin (Table S11) could be used because of their low AMR. In contrast, Trimethoprim‐Sulfamethoxazole and Ciprofloxacin prescriptions warrant carefulness.

However, the number of studies reporting MLST data was limited, reflecting the financial and technical barriers to molecular typing in Iran. Consequently, the ST distribution presented here may not fully capture the true clonal diversity of circulating *E. coli* isolates. MLST of *E. coli* isolates from Iranian studies identified five sequence types (STs): ST472 (Achtman scheme) in ESBL‐producing fecal carriage isolates, and ST131, ST77, ST506, and ST1007 (Pasteur scheme) in clinical and diarrheal settings. ST131 is by far the most epidemiologically significant. Since its initial characterization, this pandemic MDR extraintestinal pathogenic *E. coli* (ExPEC) clone has become the leading cause of community‐ and hospital‐acquired urinary tract infections (UTIs) and bloodstream infections globally (Petty et al. 2014). Its success is attributable to the co‐acquisition of CTX‐M‐15 extended‐spectrum β‐lactamase, fluoroquinolone resistance via chromosomal mutations in gyrA and parC, and an expanded virulence gene repertoire (Petty et al. 2014; Nicolas‐Chanoine et al. 2014)]. Phylogenetic analysis has delineated three major clades (A, B, C), with subclades C1/H30‐R and C2/H30‐Rx dominating MDR infections across Europe, North America, Asia, and the Middle East (Kocsis et al. 2022). Detection of CTX‐M‐15‐producing ST131 in northeast Iran aligns with data from Saudi Arabia (Alqasim et al. 2020), Turkey, India, and Europe, where ST131 accounts for 70%–80% of fluoroquinolone‐resistant isolates and up to 64% of ESBL‐producing clinical *E. coli (*Banerjee et al. 2013 *)*. The allelic profile of Iranian ST131 isolates is identical to internationally circulating strains, supporting a single clonal origin with global dispersal, consistent with prior Iranian reports documenting predominance of the H30‐Rx subclone [8].

The remaining STs ST77, ST506, and ST1007 were associated with Shiga toxin‐producing *E. coli* (STEC) from diarrheal patients and lack the pandemic lineage characteristics of ST131 (Manges et al. 2019). ST77 has been sporadically reported in STEC and enteroaggregative *E. coli* (EAEC) from Europe and Asia without evidence of clonal expansion (Pitout 2012). ST506 and ST1007 appear regionally restricted, documented primarily in the Middle East and South Asia, which may reflect genuine geographic clustering or surveillance gaps common in low‐ and middle‐income settings. ST472, recovered from fecal carriage, is present in global databases but falls outside the canonical pandemic ExPEC lineages (ST131, ST393, ST69, ST95, ST73) (Pitout 2012). Collectively, the Iranian ST distribution reflects a dual epidemiological pattern: co‐circulation of the globally dominant ST131 clone alongside regionally restricted, less‐characterized enteric lineages a phenomenon increasingly recognized across the Middle East and South Asia, where large‐scale genomic surveillance remains limited.

In animal samples, the highest resistance prevalences were observed for flumequine and erythromycin, antibiotics commonly used in veterinary medicine and food‐animal production. The low resistance to carbapenems in animals is expected, given that these agents are not approved for veterinary use; however, the observed temporal increase in resistance to chloramphenicol, trimethoprim–sulfamethoxazole, and streptomycin suggests ongoing selection driven by older or alternative antimicrobials. Conversely, declining resistance to aminoglycosides may reflect reduced usage or improved stewardship in certain animal production systems.

Environmental samples exhibited particularly high resistance to amoxicillin, tetracycline, and ampicillin, reinforcing the role of the environment as a reservoir and amplifier of AMR. The environment integrates resistant bacteria and antibiotic residues originating from hospitals, farms, wastewater, and food chains, creating conditions that favor persistence and horizontal gene transfer (Larsson and Flach 2022). The comparatively lower resistance to imipenem and cefoxitin in environmental isolates suggests that last‐resort drugs are still largely preserved outside clinical settings, though the presence of any resistance to these agents warrants close monitoring. The environment can be a reservoir of antimicrobial agents and resistant bacteria, especially through the discharge of sewage, rainwater and agricultural runoff. The environment, where it comes into contact with humans and animals, can become a platform for the transmission of resistance, and even lead to the evolution of emerging forms of resistance. This suggests that controlling AMR in the human health sector alone is not enough and that environmental dimensions should also be included in national policies and monitoring (e.g. monitoring of wastewater, impact of chemical and biological pollution) (Samreen et al. 2021). Overall, our findings demonstrate that antimicrobial resistance and resistance genes in *E. coli* have become widely established across human, animal, and environmental sectors in Iran. The high prevalence of resistance to commonly used antibiotics, together with the widespread distribution of ESBL‐producing isolates and the emerging increase in resistance to critically important antimicrobials, highlights the urgent need for continuous nationwide surveillance, coordinated antimicrobial stewardship, and evidence‐based policies to preserve the effectiveness of existing.

## 5 Conclusion

This meta‐analysis demonstrates that AMR in *E. coli* is pervasive across all One Health sectors in Iran, with particularly alarming trends in ESBL production, carbapenem resistance, and Watch‐antibiotic co‐resistance. High pooled resistance rates across Penicillins, Cephalosporins, Fluoroquinolones, and Macrolides combined with statistically significant upward temporal trends in Ertapenem, Piperacillin–Tazobactam, and Colistin define an AMR landscape that is both advanced and actively deteriorating. Fosfomycin, nitrofurantoin, amikacin, and selected carbapenems showed comparatively lower resistance and may inform the revision of empirical treatment guidelines, particularly when interpreted alongside local ESBL prevalence, clinical indications, and patient‐level factors. These findings provide the most comprehensive baseline yet available for Iran's National Action Plan (NAP‐IRIAMR), supply actionable resistance benchmarks aligned with the WHO AWaRe and IDSA 2024 frameworks, and make a compelling case for the immediate implementation of integrated, multisectoral antimicrobial stewardship programs. Sustained containment of this trajectory demands not only clinical and veterinary stewardship reform but also mandatory environmental surveillance, unified national AMR monitoring infrastructure, and a binding policy commitment to the One Health paradigm as the organizational logic for AMR governance in Iran.

Taken together, the findings support the need for an integrated One Health approach to AMR surveillance in Iran. The substantial resistance observed among human, animal, and environmental *E. coli* isolates indicates that surveillance focused exclusively on clinical settings may not fully characterize the distribution of resistance across interconnected reservoirs. Strengthening coordination among human, veterinary, and environmental surveillance systems, including harmonization of antimicrobial susceptibility testing standards, resistance‐gene characterization, and reporting practices, could improve the comparability and interpretation of AMR data. In particular, incorporating environmental surveillance alongside clinical and veterinary surveillance may provide a more comprehensive understanding of the distribution of resistant *E. coli* and resistance determinants across One Health interfaces.

## 6 Limitations

Several limitations should be considered when interpreting these findings. Grey literature was not included, which may introduce publication bias favoring higher resistance rates. The data set demonstrates a significant imbalance across One Health compartments, with human isolates overrepresented relative to animal and environmental studies, a reflection of existing surveillance inequities rather than actual epidemiological differences. Substantial heterogeneity across included studies, attributable to geographic variation, differences in patient populations and sample types, methodological inconsistencies in breakpoint criteria, and the broad 15‐year study window, limits the precision of the pooled estimates. Subgroup analyzes by sex and age were precluded by insufficient disaggregated reporting in primary studies. Finally, the predominance of hospital‐based studies may not fully capture community‐level resistance patterns. These limitations underscore the need for standardized, prospective AMR surveillance across all sectors and geographic regions in Iran. Our PROSPERO registration (CRD42023448322) originally specified a shorter search window. However, given the protracted timeline of study conduct reflecting the extensive volume of included studies, we extended the search to January 2025 to prevent the findings from becoming obsolete at the time of publication. This pragmatic adjustment, though justified, complicates comparability with the registered protocol and may have amplified heterogeneity across the 15‐year study period.

Another limitation of this study was the variability in the sources of *E. coli* isolates among the included studies. In human studies, isolates were obtained from different clinical and non‐clinical specimens, including urine, blood, stool, and other sample types, which may contribute to heterogeneity in antimicrobial resistance estimates. However, this variability reflects the One Health nature of this review, and subgroup analyses were performed according to the major reservoirs (human, animal, and environment) to partially address this issue.

## Author Contributions

**Arezoo Dehghan:** writing – original draft, writing – review and editing, data curation, investigation. **Mina Arabian:** writing – original draft, writing – review and editing, investigation, data curation. **Zahra Tahmasebi:** writing – original draft, writing – review and editing, data curation, investigation. **Aliasghar Fakhri‐Demeshghieh:** conceptualization, writing – review and editing, data curation, investigation. **Neda Baseri:** writing – review and editing, supervision, investigation. **Ehsan Mostafavi:** writing – review and editing, supervision. **Farzad Badmasti:** writing – review annd editing, methodology, supervision. **Faranak Abnaroodhelleh:** writing – review and editing, supervision. **Arash Seifi:** writing – review and editing, supervision. **Fahimeh Bagheri Amiri:** conceptualization, investigation, validation, visualization, methodology, formal analysis, supervision, software, project administration, data curation, writing – review and editing.

## Funding

The authors have nothing to report.

## Conflicts of Interest

The authors declare no conflicts of interest.

## Supporting information


**Table S1:** Pooled prevalence of E. coli isolation and antimicrobial resistance patterns in humans and animals.

## Data Availability

Data sharing not applicable to this article as no data sets were generated or analyzed during the current study.
