# Supplementary material for: Iran's Escherichia coli AMR Landscape (2010–2025): A One Health Systematic Review and Meta‐Analysis
Source: Microbiologyopen. 2026 Oct 4;15(5):e70420. doi: 10.1002/mbo3.70420 (PMC13635689; doi:10.1002/mbo3.70420)
Supplement: Supplementary file 1 [file MBO3-15-e70420-s001.docx]

**Supplement**

**Total Isolates**

The prevalence of E. coli in all studies which, had isolated E. coli from One Health samples was 38.8 (95% CI: 36.5-41.2) . Prevalence of ESBL-producing, multidrug-resistant (MDR) isolates, and AmpC-producing isolates was 47.2 (95%CI: 39.6-54.7), 64.3 (95% CI: 59.6-69.1), and 20.2 (95% CI: 15.0-25.4), respectively.

***Table S1 Pooled prevalence of E. coli isolation and antimicrobial resistance patterns in humans and animals.***

| **Source** | **Isolation of *E. coli*** |  | **MDR in *E. coli* isolates** |  | **ESBL in *E. coli* isolates** |  | **AmpC in *E. coli* isolates** |  | **MBL in *E. coli* isolates** |  |
| --- | --- | --- | --- | --- | --- | --- | --- | --- | --- | --- |
|  | % frequency (95% CI) | No. studies | % frequency (95% CI) | No. studies | % frequency (95% CI) | No. studies | % frequency (95% CI) | No. studies | % frequency (95% CI) | No. studies |
| **Human** | 37.7 (35.4–40.0) | 124 | 64.8 (58.8–70.9) | 86 | 46.2 (40.2-53.0)) | 90 | 21.7 (16.4–27.1) | 18 | 14.1% (95% CI: 9.5–18.7) | 11 |
| **Animal** | 64.4 (53.2–75.6) | 31 | 44.6 (27.1–62.2) | 21 | 11.8 (3.5–20.2) | 7 | 0.9 (0.0–2.5) | 1 | – | – |
| **Food** | 37.0 (22.4-51.6) | 19 | 72.7 (65.4-80.1) | 8 | 38.7 (25.8-51.6) | 2 | - | - | - | - |
| **Environment** | 23.4 (13.8-33.0) | 8 | 96.6 (92.9-100.0) | 4 | 34.6 (0.0-100.0) | 2 | - | - | - | - |

MDR = multidrug resistant, ESBL = extended-spectrum β-lactamase, AmpC = AmpC β-lactamase, MBL = metallo-β-lactamase

***Figure S1 Significant AMR trend of E. coli isolated from ONE-Health samples over time.***

**Classes of antibiotics**

**The prevalence of AMR of different classes of antibiotics are shown in each provinces (Table S2)**

***Table S2 The pooled prevalence of E. coli resistance to antibiotic classes, based on each province of Iran, 2010-2025.***

| **AB Classes** | **Alborz** | **Ardabil** | **Bushehr** | **Chaharmahal and Bakhtiari** | **East Azerbaijan** | **Fars** | **Gilan** | **Golestan** | **Hamadan** | **Ilam** | **Isfahan** | **Kerman** | **Kermanshah** | **Khuzestan** | **Yasuj** | **Kurdistan** | **Lorestan** | **Markazi** | **Mazandaran** | **North Khorasan** | **Qazvin** | **Qom** | **Razavi Khorasan** | **Semnan** | **Sistan and Baluchestan** | **South Khorasan** | **Tehran** | **West Azerbaijan** | **Yazd** | **Zanjan** |
| --- | --- | --- | --- | --- | --- | --- | --- | --- | --- | --- | --- | --- | --- | --- | --- | --- | --- | --- | --- | --- | --- | --- | --- | --- | --- | --- | --- | --- | --- | --- |
| **Penicillins** | 54.4(34.7- 74.1) | 65.3 (34.1-99.1) | 82.1 (75.8-88.5) | 67.8(41.1- 94.5) | 86.2(80.5- 91.8) | 76.1(52.9- 95.1) | 74(52.9- 95.1) | 77.3(47.8- 100.0) | 47.0(25.4- 68.5) | ND | 69(61.2- 76.9) | 43.8(11.6- 76) | 70.5(35.7- 100.0) | 81.9(75.2- 88.5) | 84.3(70.7- 97.8) | 71.9(56.3- 87.5) | 85.0(80.1- 89.9) | 67.5(19.1- 100.0) | 66.6(47.2- 85.9) | 84.0(73.8- 94.2) | 89.8(73.0- 100.0) | 60.9(8.4- 100.0) | 27.4(12.2- 42.7) | 48.0(0.0- 100.0) | 87.5(78.1- 96.9) | 85.0(67.6- 100.0) | 83.5(80.8- 86.2) | 68.1(28.6- 100.0) | 34.3(0- 76.9) | 81.8(71.4- 92.3) |
| **Blactam combination agents** | 35.6(18.3-52.8) | ND | ND | ND | 36.5(18.6-54.5) | 47.3(19.2-75.4) | 65.6 (46.9-84.4) | 77.3(67.9-86.8) | 45.4(11.8-79.0) | ND | 30.6(17.0-44.2) | 42.9(27.1-58.7) | ND | 39.1(26.3-51.9) | 75.1(69.8-80.3) | 12.5(1.0-24.0) |  | 50.0(40.3-59.7) | 56.8(40.6-72.9) | ND | 62.5(43.1-81.9) | 55.9(46.2-65.5) | 6.8(0.5-13.2) | 43.8(23.1-64.5) | 25.0(14.7-35.3) | 37.9(29.1-46.8) | 45.0(34.7-55.2) | 100.0(97.5-100.0) | ND | 57.3 (31.1-83.5) |
| **Cephems** | 74.1(61.8-86.4) | 26.5(0.0-55.0) | 45.0(36.8-53.2) | 45.3(31.4-59.2) | 57.8(43.2-72.3) | 55.8(46.3-65.2) | 62.5(43.6-81.4) | 42.5(0.00-86.5) | 62.4(44.8-79.9) | 90.4(78.8-100) | 57.4(44.5-70.3) | 45.7(29.9-61.5) | 56.7(42.3-71.2) | 59.6(44.4-74.8) | 84.1(77.4-90.8) | 36.6(0.00.6-75.8) | 39.7(3.5-75.9) | 51.2(31.6-70.7) | 53.7(35.8-71.7) | ND | 62.4(31.6-93.2) | 50.6(10.1-99) | 53.2(41.7-64.8) | 61.9(32.3-91.5) | 57.7(41.1-74.3) | 6.0(1.7-10.4) | 60.8(54.2-67.5) | 54.3(32.3-76.4) | 52.2(12.0-92.5) | 52.5(17.4-87.6) |
| **Monobactam** | ND | ND | ND | ND | 62.0(45.7-78.3) | 49.1(14.5-83.6) | 36.0(10.9-61.0) | ND | 23.6(8.2-39.0) | 55.7(21.8-89.5) | 63.0(47.0-79.0) | 52.4(16.7-88.0) | 25.8(17.9-33.7) | 52.4(33.9-70.9) | 77.0(65.4-88.6) | 40.6(23.6-57.6) | 60.0(50.4-69.6) | ND | 8.9(4.4-13.3) | ND | ND | ND | 15.1(5.5-24.7) | 56.7(23.9-89.5) | 44.0(34.3-53.7) |  | 41.8(29.0-54.6) | 36.1(26.9-45.2) |  | 68.8(53.2-84.5) |
| **Penems** | 6.4(2.9-9.9) | 33.3(0.0-74.8) | 29.3(21.7-36.8) | 4.4(0.0-9.9) | 2.6(1.0-4.1) | 14.0(8.2-19.8) | 19.4(13.5-25.2) | 14.0(0.0-29.5) | 17.2(12.8-21.6) | 2.0(0.0-8.1) | 2.6(1.2-3.9) | 3.8(1.7-6.0) | 3.0(1.2-4.8) | 22.5(5.8-39.2) | 2.6(0.0-5.3) | 15.7(3.2-28.2) | 0.0(0.0-1.0) | 0.0(0.0-2.0) | 13.5(8.5-18.5) | ND | 0.3(0.0-1.2) | 16.9(0.0-43.9) | 11.7(2.1-21.3) | 8.9(3.2-14.5) | 9.7(3.2-16.3) | 6.0(1.7-10.4) | 13.5(8.9-18.1) | 6.4(2.7-10.1) | 40.0(0.0-100) | 1.0(0.0-2.3) |
| **Aminoglycosides** | 36.6(12.9-60.3) | 75.0(32.6-100.0) | 20.7(14.0-27.4) | 54.6(11.4-97.9) | 32.0(20.9-43.0) | 33.9(22.4-45.4) | 35.9(23.6-48.2) | 16.3(0.0-38.8) | 21.2(15.2-27.2) | 28.2(0.0-57.1) | 34.1(25.8-42.4) | 26.9(19.6-34.1) | 27.2(15.8-38.5) | 36.7(23.5-50.0) | 27.9(2.5-53.3) | 39.6(26.4-52.7) | 31.9(17.9-45.9) | 12.2(0.0-26.2) | 29.5(20.3-38.7) | 22.0(10.5-33.5) | 29.8(2.9-56.8) | 19.9(0.9-39.0) | 30.1(2.3-57.9) | 35.4(20.6-50.2) | 23.2(14.3-32.1) | 65.5(0.0-100) | 34.9(29.1-40.6) | 38.0(29.2-46.9) | 15.7(6.0-25.4) | 33.5(14.0-53.0) |
| **Folat pathway A** | 28.3(17.9-38.8) | 56.4(6.2-100.0) | 57.9(49.7-66.0) | 49.7(18.2-81.3) | 56.2(44.1-68.3) | 54.2(47.3-61.0) | 60.1(42.5-77.7) | 25.4(0.0-56.4) | 49.1(38.5-59.6) | 61.1(31.4-90.8) | 45.4(36.2-54.6) | 53.1(35.6-70.6) | 57.3(24.0-90.6) | 60.2(45.8-74.7) | 65.0(61.4-68.7) | 68.4(59.4-77.4) | 67.9(58.7-77.1) | 64.4(56.8-72.1) | 55.9(42.8-69.1) | 60(46.4-73.6) | 43.6(0.0-95.6) | 50.9(15.9-85.8) | 34.1(17.0-51.3) | 72.3(68.5-76.0) | 69.3(65.4-73.3) | 20.7(2.7-38.7) | 59.0(51.4-66.5) | 59.1(33.1-85.1) | 68.9(64.3-73.6) | 40.0(13.5-66.6) |
| **Fosfomycins** | ND | ND | ND | ND | 15.6(4.6-26.6) | ND | 26.3(6.3-46.2) | ND | ND | ND | 27.0(23.0-31.1) | ND | 0.0(0.0-0.5) | 4.5(0.0-9.7) | ND | ND | ND | ND | ND | ND | 1.1(0.0-3.3) | ND | ND | 0.0(0.0-1.4) | 1.6(0.0-3.2) | ND | 2.9(1.1-4.7) | ND | 17.9(12.8-23.1) | ND |
| **Glycopeptides** | ND | ND | ND | 31.3(8.5-54.0) | ND | 0.0 (0.0 -4.6) | ND | ND | ND | ND | ND | ND | ND | ND | ND | ND | ND | ND | ND | ND | ND | ND | ND | ND | ND | ND | 0.0(0.0-2.3) | ND | 100(98.1-100) | ND |
| **Lincosamides** | ND | 63.2(0.0-100.0) | ND | 34.0(20.9-47.1) | ND | 56.9(0.0-100.0) | 6.6(2.2-10.9) | 50.0(21.7-78.3) | ND | ND | 30.0(17.3-42.7) | ND | 0.0(0.0-0.5) | 17.2(11.7-22.7) | ND | ND | ND | ND | ND | ND | ND | ND | 100.0(97.1-100.0) | ND | ND | 18.3(0.0-51.2) | 2.4(0.0-9.3) | ND | ND | ND |
| **Lipopeptides** | ND | 61.5(39.9-83.1) | ND | 31.3(8.5-54.0) | 49.5(40.2-58.9) | 0.2(0.0-1.1) | 9.4(0.0-19.5) | 2.0(0.0-1.6) | 5.6(2.9-8.4) | ND | 41.8(11.5-72.1) | 1.4(0.0-3.2) | 0.0(0.0-0.5) | 8.0(3.9-12.1) | ND | ND | ND | ND | 33.3(0.0-71.1) | ND | 16.7(1.8-31.6) |  | ND | ND | 22.0(13.9-30.1) | ND | 11.1(0.0-31.6) | ND | 8.6(0.0-26.1) | ND |
| **Macrolides** | ND | 86.3(80-92.5) | ND | 20.0(8.9-31.1) | 53.4(21.7-85.1) | 85.5(74.6-96.4) | 49.5(40.0-59.1) | ND | 35.8(7.7-63.9) | ND | ND | 69.5(8.6-100.0) | 68.5(40.4-96.6) | 59.9(21.6-98.1) | ND | ND | ND | 25.4(16.2-34.6) | 62.3(55.1-69.4) | ND | 0.0(0.0-5.5) | 41.6 (37.9-45.3) | ND | ND | 57.2(52-62.4) | 10.3(4.8-15.9) | 62.8(42.2-83.3) | 50.0(36.7-63.3) | 83.1(78.0-88.1) | 50.0(0.0-100.0) |
| **Nitroheterocyclics** | 6.2(3.4-9.1) | 26.8(0.0-83.2) | 1.4(0.0-3.4) | 15.5(5.0-26.1) | 15.1(3.9-26.3) | 19.9(11.7-28.0) | 21.5(0.0-43.7) | 38.3(19.4-57.1) | 15.0(9.4-20.6) | 0.0(0.0-3.4) | 18.8(11.9-25.7) | 5.0(2.1-7.9) | 16.8(9.3-24.4) | 16.6(8.7-24.5) | 0.7(0.0-2.1) | 3.1(0.4-5.7) | 3.2(1.1-5.3) | 3.7(0.0-11.2) | 18.6(11.2-26.1) | 6.0(0.0-12.6) | 28.3(0.0-77.0) | 11.3(5.4-17.3) | 7.6(2.4-12.8) | 9.6(3.2-16.0) | 14.0(7.5-20.5) | ND | 12.2(9.1-15.3) | 19.6(1.8-37.4) | 7.3(3.8-10.8) | 16.8(11.5-22.1) |
| **Phenicols** | 17.6(13.0-22.3) | 47.1(1.3-92.8) | ND | 50.0(0.0-100.0) | 28.2(19.0-37.5) | 26.5(9.7-43.3) | 35.3(12.9-57.7) | ND | 37.7(8.0-67.5) | ND | 27.1(14.3-39.9) | 19.1(4.2-34.0) | 0.0(0.0-0.5) | 7.0(0.4-13.6) | 11.9(7.1-16.7) | 28.6(9.2-47.9) | ND | 56.5(45.9-67) | 15.8(0.0-32.2) | ND | 54.2(34.2-74.1) | ND | 13.8(6.4-21.3) | ND | ND | 34.0(0.0-92.2) | 32.2(13.4-50.9) | ND | 71.3(65.2-77.3) | 0.0(0.0-1.9) |
| **Quinolones** | 37.8(24.4-51.1) | 62.2(20.9-100.0) | 45.0(36.8-53.2) | 50.3(16.7-84.0) | 49.6(37.0-62.2) | 50.1(39.8-60.4) | 56.4(41.7-71.0) | 29.5(0.0-70.8) | 53.4(36.2-70.7) | 60.6(7.9-100.0) | 49.9(43.3-56.5) | 44.3(29.2-59.3) | 37.6(17.5-57.6) | 35.7(23.2-48.2) | 54.2(48.0-60.4) | 65.2(46.5-83.9) | 63.0(40.7-85.3) | 75.3(54.7-96) | 42.9(23.7-62.2) | 60.0(46.4-73.6) | 28.7(0.0-67.8) | 48.5(12.4-84.5) | 23.6(6.8-40.3) | 64.9(47.7-82.1) | 50.1(44.4-55.9) | ND | 55.5(48.1-62.9) | 63.9(43.7-84.1) | 80.2(70.3-90.2) | 45.1(23.8-66.4) |
| **Tetracyclines** | 55.7(43.6-67.8) | 59.6(0.0-100.0) | ND | 73.4(27.7-100) | 54.3(45.6-63.0) | 54.6(30.3-79.0) | 68.3(50.5-86.1) | 14.7(6.7-22.7) | 67.7(51.9-83.5) | ND | 66.3(53.5-79.1) | 57.0(34.4-79.7) | 29.8(3.9-55.8) | 58.1(47.2-69.1) | 66.2(43.5-89.0) | 62.5(45.7-79.3) | 66.0(56.7-75.3) | 80.5(72.1-88.9) | 61.8(25.2-98.4) | ND | 54.2(34.2-74.1) | 0.6(0.0-1.8) | 42.3(26.5-58.2) | 74.0(65.4-82.6) | 57.0(47.3-66.7) | ND | 57.5(45.2-69.8) | 68.5(56.1-81.0) | 54.8(48.1-61.4) | 53.6(22.4-84.8) |

\# Red color prevalence is not driven using meta-analysis, ND: No data

***Table S3 The pooled AMR prevalence of E. coli to antibiotic classes, based on time-points, 2010-2025, Iran.***

|  | Total Prevalence (95% CI) | 2009-2015(95% CI) | 2016-2019(95% CI) | 2020-2025(95% CI) | P Value | Difference (95%CI) |
| --- | --- | --- | --- | --- | --- | --- |
| Penicillins | 71.7(65.6-76.6) | 71.5(66.3-76.8) | 71.7(67.1-76.4) | 69.6(52.1-87.2) | 0.82 | -0.70 (-6.89, 5.48) |
| B-lactam combination agents | 44.7(6.4-50.6) | 47.7(39.3-56.2) | 42.4(31.1-53.6) | 41.7(31.9-51.4) | 0.41 | -3.24(-10.9,4.52) |
| Cephems | 57.3(53.6-61.1) | 58.5(53.8-63.2) | 56.9(50.5-63.3) | 55.9(46.4-65.3) | 0.60 | -1.29(-6.09,3.50) |
| Monobactam | 47.9(40.9-54.8) | 46.2(35.1-57.3) | 46.2(36.2-56.2) | 55.8(36.7-75.0) | 0.43 | 3.87(-5.63, 13.36) |
| Penems | 12.8(11.0-14.6) | 10.7(8.5-12.9) | 13.6(10.8-16.5) | 15.5(7.4-23.6) | 0.10 | 1.85(-0.54,4.37) |
| Aminoglycosides | 33.1(30.2,35.9) | 38.9(33.9-43.9) | 29.2(24.9-33.5) | 27.9(21.1-34.8) | 0.002 | -6.12(-10.0,-2.21) |
| Folat pathway A | 53.8(49.2-58.4) | 49.6(43.8-55.4) | 57.3(50.8-63.8) | 55.9(45.7-66.2) | 0.20 | 4.17(-1.36,9.71) |
| Fosfomycins | 6.8(5.2-8.5) | 21.2(6.4-35.9) | 6.8(4.2-9.4) | 3.0(1.3-4.6) | 0.00 | -6.22(-8.80,-3.64) |
| Glycopeptides | 26.5(0.0-84.8) | 9.6(0.0-30.4) | 50.0(0.0-100.0) | - | - | - |
| Lincosamides | 31.5(18.0-44.9) | 18.8(8.7-28.9) | 70.0(30.3-100.0) | 9.0(3.9-14.2) | 0.80 | 2.00(-13.25,17.27) |
| Lipopeptides | 10.5(8.4-12.6) | 11.1(7.5-14.6) | 12.0(6.4-17.7) | 16.7(6.8-26.6) | 0.91 | 0.7(-2.83,4.23) |
| Macrolides | 58.9(46.9-71.0) | 49.7(23.7-75.7) | 62.2(43.1-81.4) | 61.9(44.7-79.2) | 0.49 | 5.35(-12.15,22.86) |
| Nitroheterocyclics | 13.5(12.0-15.0) | 12.3(10.3-14.4) | 15.8(12.8-18.8) | 11.1(8.2-14.0) | 0.78 | 0.32(-1.77,2.40) |
| Phenicols | 28.6(24.1-33.1) | 24.9(17.0-32.8) | 33.3(24.4-42.2) | 30.6(21.7-39.5) | 0.19 | 4.43(-1.87,10.73) |
| Quinolones | 50.0(45.4-54.6) | 48.8(43.3-54.4) | 52.1(45.4-58.7) | 48.4(39.9-56.9) | 0.92 | 0.76(-5.10,6.60) |
| Tetracyclines | 58.9(52.0-65.9) | 60.2(54.1-66.3) | 60.6(47.7-73.4) | 53.0(38.7-67.4) | 0.47 | -2.99(-10.57,4.59) |

***Figure S2 Percent of AMR for AB classes based human samples; A: Penicillins, B: B-lactam combination agents, C: Cephems, D: Monobatams, E: Penems*, F: Aminoglycosides*,G: Folate pathway antagonists", H: Fosfomycins*, I: Lincosamides, J: Lipopeptides*, K: Macrolides, L: Phenicols, M: Quinolones, N: Tetracyclines***

***Figure S3 Percent of AMR for AB classes based animal samples: A: Penicillins, B: B-lactam combination agents, C: Cephems, D: Monobatams, E: Penems, F: Aminoglycosides, G: Folate pathway antagonists, H: Fosfomycins, I: Lincosamides, J: Lipopeptides, K: Macrolides, L: Phenicols*, M: Quinolones, N: Tetracyclines***

***Figure S4 Percent of AMR for AB classes based environmental samples: A: Penicillins*, B:B-lactam combination agents, C: Cephems, D: Penems, E: Aminoglycosides, F: Folate pathway antagonists, G: Glycopeptides, H: Lincosamides, I: Lipopeptides, J: Macrolides, K: Phenicols, L: Quinolones, M: Tetracyclines***

**Antibiotic resistance results in human samples**

Human samples included clinical samples such as blood, CSF, stool, urine, respiratory secretions, vaginal cultures, wounds, extra-intestinal samples, tracheal aspirates, sputum, biopsy samples, etc.

***Table S4 Pooled prevalence of AMR of E. coli and meta-regression (between group and during time-point) in human samples, 2010-2025, Iran.***

| AWaRe | Antibiotics | No of study | Total | Healthy people | Pathogenic | Patients with diarrhea | UTI patients | Meta-regression Time-point | Meta-regression Within groups |
| --- | --- | --- | --- | --- | --- | --- | --- | --- | --- |
| Access | **Amoxicillin/Clavulanic acid** | 68 | 51.4(44.9-57.9) | 51.4(24.3-78.6) | 52.2(40.8-63.6) | 49.4(30.4-68.4) | 51.3(41.9-60.8) | -5.8(-14.7 to 3.1) | -0.6(-6.4 to 5.3) |
|  | **Amikacin** | 161 | 16.8(13.7-19.9) | 7.1(3.1-11.1) | 22.1(13.9-30.3) | 17.2(12.1-22.3) | 11.7(10.1-13.3) | -1.0(-5.1 to 3.1) | -2.7(-5.7 to 0.2) |
|  | **Amoxicillin** | 40 | 75.5(68.5-82.5) | 70.5(57.6-83.3) | 65.6(43.9-87.4) | 78.8(68.8-88.8) | 81.9(76.2-87.7) | 0.8(-7.9 to 9.4) | 6.2(0.15 to 12.1) |
|  | **Ampicillin** | 124 | 74.9(71.4-78.4) | 48.8(30.8-66.9) | 78.5(70.4-86.7) | 70.7(61.0-80.4) | 77.1(73.8 to 1.2) | 4.5(-0.7 to 9.6) | 3.1(-0.8 to 6.9) |
|  | **Chloramphenicol** | 42 | 23.7(17.2-30.2) | 7.5(3.7-11.3) | 19.6(11.3-27.8) | 30.0(19.4-40.7) | 24.9(12.3-35.7) | 1.9(-8.5 to 12.2) | 4.1(-3.0 to 11.1) |
|  | **Cephalothin** | 31 | 60.9(53.2-68.5) | 47.8(0.0-100.0) | 51.9(38.6-65.2) | 44.5(0.0-93.6) | 66.7(59.0-74.3) | -4.5(-15.2 to 6.2) | 6.5(0.4 to 12.5) |
|  | **Cephalexin** | 24 | 63.9(54.5-73.3) | 26.2(4.6-47.8) | 80.7(69.2-92.2) | 65.1(56.0-74.2) | 59.4(48.0-70.7) | -1.2(-16.3 to 13.9) | 0.67(-11.3 to 12.7) |
|  | **Cefazolin** | 47 | 65.4(58.3-72.5) | 32.0(0.0-86.1) | 67.2(56.6-77.9) | 97.5(92.7-100.0) | 64.9(55.6-74.3) | -4.4(-13.1 to 4.4) | -1.1(-11.9 to 9.7) |
|  | **Doxycycline** | 12 | 57.4(33.3-81.4) | 35.7(0.0-78.8) | 61.9(47.6-76.3) | - | 61.1(7.1-100.0) | 4.2(-29.8,38.2) | 10.2(-25.7 to 46.1) |
|  | **Nitrofurantoin** | 117 | 11.2(9.8-12.5) | 0.5(0.0-3.0) | 13.3(10.6-15.9) | 4.9(1.6-8.3) | 11.0(9.3-12.6) | 0.6(-1.2 to 2.4) | 1.8 (0.4 to 3.2) |
|  | **Gentamicin** | 214 | 29.0(26.3-31.6) | 10.3(6.1-14.4) | 36.0(30.0-42.0) | 20.9(15.4-26.5) | 27.9(24.7-31.1) | -6.7(-10.3 to -3.2) | 3.3(1.7 to 4.9) |
|  | **Penicillin** | 9 | 99.6(98.5-100.0) | 92.1(87.4-96.8) | 100.0(90.5-100.0) | 85.1(49.2-100.0) | 100.0(99.5-100.0) | - | - |
|  | **Ampicillin/SULBACTAM** | 22 | 41.4(29.8-53.0) | - | 40.0(25.5-54.5) | 34.6(7.4-61.7) | 43.6(24.5-62.8) | 1.9(-14.3 to 18.3) | 3.1(-10.1 to 16.2) |
|  | **Sulfamethoxazole** | 5 | 48.8(38.5-59.1) | 33.3(16.5-50.2) | 40.0(30.4-49.6) | - | 56.1(44.4-67.7) | - | - |
|  | **Trimethoprim- Sulfamethoxazole** | 197 | 57.9(54.4-61.4) | 42.4(23.5-61.2) | 56.5(50.7-62.3) | 55.1(42.5-67.8) | 60.9(56.7-65.1) | 4.3(-0.3,9.0) | 3.1(-0.3 to 6.5) |
|  | **Tetracycline** | 102 | 58.2(50.0-66.5) | 44.0(31.5-56.5) | 60.8(42.5-79.1) | 51.0(37.5-64.5) | 62.9(56.3-69.5) | -0.5(-11.8 to 10.8) | 3.8(-3.3 to 10.7) |
|  | **Sulphadiazine+trimethoprim** | 4 | 63.4(51.2-75.5) | - | - | 37.5(4.0-71.0) | 66.1(53.7-78.5) | - | - |
|  | **Trimethoprim** | 12 | 59.2(51.2-67.2) | 81.0(73.3-88.7) | 56.1(49.7-62.4) | 56.9(32.3-81.5) | 57.7(47.4-67.9) | -3.8(-13.8 to 6.3) | -1.2(-8.2 to 5.7) |
| Watch | **Azithromycin** | 16 | 46.3(32.3-60.3) | - | 43.6(18.1-69.2) | 42.6(21.0-64.2) | 52.9(26.5-79.4) | -1.2(-22.0 to 19.7) | 3.1(-8.7 to 15.0) |
|  | **Carbenicillin** | 2 | 51.9(32.4-71.4) | - | 51.9(32.4-71.4) | - | - | - | - |
|  | **Ceftazidime** | 181 | 47.1(42.0-52.2) | 34.6(21.3-47.9) | 52.0(41.3-62.8) | 43.0(32.0-54.0) | 46.3(42.0-50.6) | 4.4(-1.9 to 9.9) | 3.9(0.8 to 8.6) |
|  | **Cefixime** | 61 | 50.4(44.9-55.8) | 40.0(26.4-53.6) | 57.5(43.4-71.6) | 52.8(38.0-67.6) | 45.8(40.2-51.5) | 4.3(-4.4 to 13.1) | -5.0(-10.9 to 0.1) |
|  | **Ciprofloxacin** | 234 | 41.5(37.9-45.0) | 35.6(17.9-53.2) | 46.1(39.3-52.8) | 21.7(16.1-27.2) | 44.5(40.2-48.9) | 4.8(0.07 to 9.61) | 4.7(2.6 to 6.8) |
|  | **Cefpodoxime** | 19 | 66.4(45.1-87.7) | - | 67.9(56.1-79.6) | 52.4(0.0-100.0) | 72.4(60.4-84.4) | 4.2(-22.8 to 31.2) | 2.4(-22.9 to 27.6) |
|  | **Ceftriaxone** | 114 | 50.1(43.8-56.5) | 28.6(15.9-41.4) | 58.6(50.8-66.4) | 43.2(27.1-59.3) | 49.4(41.6-57.3) | 6.5(-1.4 to 14.5) | 3.5(0.4 to 6.6) |
|  | **Cefuroxime** | 34 | 56.3(42.3-70.3) | 29.4(0.0-89.8) | 62.9(47.0-78.7) | 42.6(20.4-64.8) | 57.6(38.1-77.1) | 0.1(-17.6 to 17.8) | 1.1(-13.2 to 15.3) |
|  | **Cefotaxime** | 182 | 53.7(48.3-59.0) | 43.2(22.5-64.0) | 58.4(50.2-66.5) | 43.8(32.9-54.6) | 54.4(47.4-61.4) | 7.7(1.2 to 14.3) | 0.5(-3.4 to 5.0) |
|  | **Ceftizoxime** | 21 | 38.5(29.5-47.5) | 4.0(0.0-9.4) | 50.1(35.9-64.4) | 36.7(0.0-78.0) | 30.5(18.1-42.9) | 16.7(-4.1 to 3.75) | -6.0(-15.0 to 3.1) |
|  | **Doripenem** | 6 | 3.8(0.9-6.7) | - | 7.4(1.9-12.8) | - | 0.0(0.0-1.2) | - | - |
|  | **Erythromycin** | 12 | 89.1(84.8-93.5) | 94.4(87.6-100.0) | 92.7(86.6-98.7) | 81.9(44.9-100.0) | 63.9(54.7-73.1) | -5.9(-1.9 to -0.93) | -3.6(-5.5 to -1.7) |
|  | **Enrofloxacin** | 2 | 22.4(0.8-44.0) | 33.6(25.1-42.1) | 11.6(5.1-18.0) | - | - | - | - |
|  | **Ertapenem** | 23 | 4.9(3.5-6.4) | - | 13.7(9.5-18.0) | 0.6(0.0-1.7) | 1.0(0.1-1.8) | 3.9(1.4 to 6.5) | -3.4(-5.3 to -1.6) |
|  | **Cefepime** | 69 | 42.9(35.1-50.6) | 47.4(6.9-87.9) | 46.7(35.6-57.8) | 38.3(18.2-58.4) | 39.7(26.9-52.5) | 0.03(-11.9 to 12.2) | -3.3(-11.2 to 4.6) |
|  | **Cefoxitin** | 55 | 31.6(24.4-34.5) | 24.7(0.0-50.3) | 35.6(25.6-45.6) | 31.0(0.9-61.0) | 29.5(20.0-39.1) | -1.2(-9.9 to 7.5) | -1.1(-10.1,8.8) |
|  | **Gatifloxacin** | 3 | 59.9(53.9-65.8) | - | 53.2(38.9-67.5) | - | 61.1(54.2-68.0) | - |  |
|  | **Kanamycin** | 16 | 18.1(12.8-23.5) | 9.4(3.0-15.9) | 25.1(9.6-40.7) | 15.0(5.2-24.8) | 21.4(13.3-29.5) | 8.1(0.7 to 15.4) | 3.6(-0.02 to 7.2) |
|  | **Levofloxacin** | 35 | 38.3(30.0-46.7) | 6.0(0.0-12.6) | 48.5(28.0-69.1) | 12.3(1.0-23.6) | 41.6(33.2-49.9) | 3.1(-8.2 to 14.3) | 7.2(1.9 to 12.4) |
|  | **Meropenem** | 73 | 8.0(5.6-10.4) | 3.0(0.0-6.4) | 13.8(8.0-19.7) | 0.6(0.0-1.5) | 1.9(1.1-2.6) | 2.4(-1.0 to 5.9) | -3.1(-5.5 to -0.7) |
|  | **Minocycline** | 3 | 29.0(21.9-36.1) | - | 28.8(19.8-37.7) | 30.0(13.6-46.4) | - | - | - |
|  | **Neomycin** | 3 | 34.4(12.3-56.6) | 16.0(5.8-26.2) | - | - | 43.6(18.6-68.5) | - | - |
|  | **Netilmicin** | 2 | 26.3(22.3-30.3) | - | 25.3(21.4-29.3) | - | 30.6(21.5-39.7) | - | - |
|  | **Norfloxacin** | 43 | 38.1(31.8-44.4) | 6.0(0.7-11.3) | 39.4(22.0-56.8) | - | 42.8(31.4-54.1) | -2.2(-13.7 to 4.7) | 6.7(0.3 to 13.1) |
|  | **Ofloxacin** | 34 | 41.5(36.0-47.1) | 20.0(0.0-44.8) | 50.3(42.1-58.4) | 4.3(0.0-12.7) | 40.6(34.5-46.7) | 4.1(-5.1 to 13.2) | -2.2(-8.2 to 3.6) |
|  | **Oxytetracycline** | 2 | 54.5(7.3-100.0) | 78.2(70.7-85.6) | 30.0(15.8-44.2) | - | - | - | - |
|  | **Piperacillin** | 34 | 49.5(36.6-62.4) | 0.0(0.0-2.7) | 51.8(34.3-69.2) | 71.8(57.7-85.9) | 48.9(34.7-63.0) | 4.1(-13.5 to 21.7) | 1.5(-11.5 to 14.4) |
|  | **Streptomycin** | 16 | 33.4(24.3-42.4) | 29.5(14.0-45.0) | - | 38.3(22.3-54.3) | 35.4(22.1-48.7) | 12.8(-1.3 to 24.9) | 2.9(-3.3 to 9.2) |
|  | **Piperacillin- Tazobactam** | 49 | 16.6(13.9-19.4) | 13.0(6.4-19.6) | 18.0(13.6-22.4) | 4.3(0.0-16.2) | 16.6(12.3-20.9) | 2.6(-1.1 to 6.4) | -0.01(-3.1 to 3.0) |
| Reserve | **Aztreonam** | 61 | 48.8(41.8-55.9) | 62.7(27.0-98.3) | 57.7(47.7-67.8) | 44.1(22.9-65.3) | 42.3(33.4-51.3) | 4.7(-4.7 to 14.1) | -7.4(-13.6 to -1.1) |
|  | **Colistin** | 2 | 2.0(0.8-3.1) | 0.0(0.0-1.4) | 3.2(1.1-5.2) | 7.7(0.0-22.2) | 3.1(0.0-7.4) | - | - |
|  | **Tigecycline** | 6 | 15.4(8.9-21.9) | - | 15.4(8.9-21.9) | - | - | - | - |
|  | **Fosfomycin** | 17 | 6.3 (3.9-8.6) |  | 5.7 (2.1-9.3) | - | 7.8 (3.8-11.8) | -0.04 (-0.08 to 0.02) | -0.01 (-0.04 to 0.03) |
| Not in AWaRe | **Imipenem** | 185 | 11.8(10.2-13.4) | 5.2(2.0-8.4) | 12.2(10.5-13.9) | 13.6(4.9-22.4) | 9.9(7.7-12.2) | 2.4(0.2 to 4.7) | -0.9(-2.6 to 0.6) |
|  | **Nalidixic acid** | 123 | 57.6(53.0-62.2) | 50.8(20.5-81.1) | 51.7(41.4-62.1) | 60.4(46.6-74.3) | 60.0(54.7-65.3) | 1.1(-5.8 to 8.0) | 3.6(-1.1 to 8.2) |
|  | **Ticarcillin/clavulanic acid** | 2 | 66.2(57.0-75.4) | - | 62.0(57.6-66.4) | 71.4(63.9-78.9) | - | - | - |

***Figure S 5 Significant AMR trend of E. coli isolated from human population during time-points, 2010-2025, Iran***

**Antibiotic resistance in animal samples**

Animal samples were included: tissue, fecal and rectal swab, milk, insect, etc. Animal species were included: healthy (asymptomatic) animals and animals with symptoms of broiler, sheep, goat, cattle, buffaloes, cockroaches, flies, rats, ostrich, pigeon, horse, pet cats, and dogs.

**Table S 5 Pooled prevalence of AMR of E. coli and meta-regression (between group and during time-point) in animal samples, 2010-2025, Iran.**

| **AWaRe** | **Abbreviations** | **No of study** | **Total** | **Chicken** | **Insect** | **Other** | **Ruminant** | **Meta-regression** | |
| --- | --- | --- | --- | --- | --- | --- | --- | --- | --- |
|  |  |  |  |  |  |  |  | **Time-point** | **Group** |
| **Access** | **AMC** | 15 | 24.2(16.54-31.84) | 20.5(13.0-28.0) | - | 43.7(17.6-69.8) | 14.6(6.9-22.4) | 7.7(-2.8 to 18.3) | -3.1(-11.1 to 4.9) |
|  | **AN** | 13 | 14.9(10.6-19.3) | 16.4(9.0-23.8) | 4.8(0.2-9.3) | 24.6(14.1-35.1) | 17.4(6.3-28.6) | -13.5(-22.3 to -4.7) | 1.1(-4.6 to 6.6) |
|  | **AMX** | 11 | 47.6(18.7-76.5) | 58.8(7.3-100.0) | 89.9(84.5-95.3) | 40.0(23.8-56.2) | 36.3(2.5-70.1) | -21.1(-63.9 to 21.8) | -10.5(-34.6 to 13.7) |
|  | **AM** | 30 | 49.7(35.8-63.7) | 43.7(22.3-65.2) | 54.6(0.0-100.0) | 62.2(42.9-81.6) | 45.7(17.9-73.4) | 1.9(-19.5 to 23.4) | 1.1(-12.3 to 14.5) |
|  | **C** | 24 | 36.5(26.5-46.5) | 56.9(36.3-77.4) | 2.0(0.0-6.0) | 44.8(25.7-63.9) | 16.8(9.9-23.7) | 12.6(3.6 to 21.6) | -10.4(-16.7 to -4.2) |
|  | **CF** | 5 | 36.5(4.3-68.7) | 31.3(23.9-38.8) | 2.0(0.0-6.0) | - | 74.0(55.1-93.0) | - | - |
|  | **CN** | 6 | 8.2(5.2-11.1) | 8.5(3.7-13.4) | - | - | 8.2(3.4-13.1) | - | - |
|  | **CZ** | 4 | 6.1(1.5-10.7) | 3.3(0.5-6.2) | - | 18.5(9.0-27.9) | 4.7(-2.5-11.9) | - | - |
|  | **DO** | 15 | 46.7(25.4-68.0) | 57.2(35.0-79.5) | - | 69.7(48.2,91.1) | 26.0(0.0-60.5) | 10.0(-21.0 to 41.0) | -10.0(-26.6 to 6.5) |
|  | **FM** | 8 | 22.2(2.6-41.9) | 36.8(0.0-83.7) | 21.2(14.9-27.4) | 12.3(3.6-20.9) | 0.0(0.0-5.5) |  | - |
|  | **GM** | 37 | 14.2(7.6 to 20.7) | 13.5(3.2-23.8) | 17.3(0.0-36.1) | 5.2(1.7-8.5) | 15.8(1.4-30.2) | -11.8(-21.2 to -2.5) | -0.8(-6.6 to 5.1) |
|  | **OX** | 2 | 48.5(8.0-89.0) | 69.2(54.7-83.7) | - | - | 27.9(14.5-41.3) | - | - |
|  | **P** | 4 | 83.3(58.9-100.0) | 45.9(36.7-55.2) | - | 94.2(85.3-100.0) | 96.9(92.6-100.0) | - | - |
|  | **SMX** | 3 | 49.3(0.0-98.9) | 72.6(66.4-78.7) | - | - | 3.2(0.0-9.4) | - | - |
|  | **SXT** | 36 | 39.0(30.3-47.7) | 64.0(36.5-91.4) | 25.5(0.0-55.9) | 35.6(23.6-47.7) | 21.0(13.3-28.7) | 14.1(6.1 to 22.1) | -12.1(-16.5 to -7.5) |
|  | **TE** | 27 | 49.7(34.1-65.3) | 68.8(36.4-100.0) | 33.3(0.0-71.5) | 52.7(30.8-74.6) | 43.9(31.2-56.7) | 2.1(-12.0 to 16.2) | -5.9(-15.7 to 3.9) |
| **Watch** | **AZM** | 3 | 12.5(6.5-18.6) | 10.3(4.8-15.9) | - | 21.7(9.8-33.7) | 10.0(0.7-19.3) | - | - |
|  | **CAZ** | 18 | 15.7(9.9-21.6) | 2.3(0.7-3.9) | 70.4(49.5-91.2) | 15.0(1.6-18.1) | 7.2(2.7-11.8) | -8.3(-17.7 to 1.0) | 0.7(-5.4 to 6.8) |
|  | **CFM** | 8 | 24.7(0.2-49.2) | 26.0(0.0-59.3) | - | 22.2(0.0-49.4) | 22.9(14.8-30.9) | - | - |
|  | **CIP** | 28 | 33.0(24.5-42.1) | 56.8(29.8-83.7) | 37.1(-5.9-80.2) | 30.5(11.7-49.3) | 7.0(3.3-10.6) | 3.2(-6.9 to 13.3) | -14.5(-20.7 to -8.3) |
|  | **CRO** | 14 | 18.1(12.9-23.2) | 9.1(3.7-14.5) | 78.6(69.8-87.3) | 25.6(0.0-54.3) | 8.3(0.0-18.9) | 2.6(-7.7 to 12.9) | -14.7(-21.0 to -8.4) |
|  | **CTC** | 3 | 65.3(0.0-100.0) | 98.3(94.3-100.0) | - | - | 0.0(0.0-0.5) | - |  |
|  | **CTX** | 24 | 23.6(0.0-49.1) | 6.7(4.8-8.5) | 50.0(15.7- 84.4) | 27.3(13.8-40.9) | 15.1(7.5-22.8) | -4.5(-13.3 to 4.2) | 2.0(-3.3 to 7.3) |
|  | **ZOX** | 3 | 19.5(3.4-35.6) | 3.3(0.5-6.2) | 57.1(8.3-66.0) | - | 1.3(0.0-3.2) | - | - |
|  | **E** | 13 | 61.1(37.2-85.0) | 77.7(69.5-85.9) | 28.3(15.2-41.3) | 74.4(45.8-100.0) | 31.2(3.0-59.4) | 21.0(-11.1 to 52.8) | -11.7(-34.6 to 11.3) |
|  | **FEP** | 5 | 22.5(0.0-55.0) | 9.3(0.0-22.5) | 69.0(59.2-78.9) | 0.0(0.0-12.3) | - | - | - |
|  | **FOX** | 5 | 15.9(4.4-27.5) | 65.7(50.0-81.4) | - | 8.7(0.6-16.8) | 5.7(0.0-13.9) | - | - |
|  | **FLU** | 8 | 62.3(19.1-100.0) | 88.8(82.7-94.9) | - | 52.1(33.8-70.4) | 0.0(0.0-0.5) | - | - |
|  | **K** | 8 | 18.9(11.0-26.9) | 5.3(1.7-8.9) | 19.6(8.1-31.0) | 23.8(4.0-43.5) | 24.2(0.5-47.8) | - | - |
|  | **LIN** | **1** | 100.0(97.1-100.0) | - | - | - | 100.0(97.1-100.0) | - | - |
|  | **LVX** | 7 | 15.8(6.7-24.9) | 17.3(0.0-37.5) | - | 18.4(6.6-30.1) | 14.9(0.0-34.0) | - | - |
|  | **MEM** | 5 | 1.8(0.0-3.91) | 1.8(0.0-4.3) | - | 2.2(0.0-6.4) | - | - | - |
|  | **NEO** | 12 | 31.1(5.4-52.7) | 32.9(4.0-61.9) | - | 26.6(19.6-33.7) | 31.1(0.0-83.8) | -1.4(-16.1 to 13.2) | -1.1(9.7 to 7.4) |
|  | **NX** | 8 | 9.1(4.8-13.4) | 15.3(9.6-21.1) | 38.7(29.9-47.7) | 11.1(0.0-31.6) | 1.9(0.0-3.9) | - | - |
|  | **OTC** | 12 | 50.2(27.7-72.8) | 82.3(51.7-100.0) | - | 64.8(57.7-71.8) | 25.2(16.5-33.9) | 4.9(-13.1 to 23.0) | -19.4(-27.5 to -11.3) |
|  | **PIP** | 4 | 1.5(0.0-4.7) | 0.7(0.0-2.0) | 55.6(23.1-88.0) | - | 11.9(0.0-38.8) | - | - |
|  | **STR** | 18 | 31.5(0.7-62.3) | 4.7(1.3-8.0) | - | 35.0(18.1 to 52.0) | 25.3(14.8-35.8) | 19.4(11.2 to 27.5) | 1.2(-6.3 to 8.7) |
|  | **TOB** | 4 | 27.1(13.5-40.7) | 18.2(5.3-31.1) | - | - | 40.4(33.0-47.8) | - | - |
| **Reserve** | **CL** | 11 | 14.4(10.3-18.5) | 22.6(13.7-31.5) | - | 8.4(3.9-12.8) | 24.4(0.0-73.4) | -3.8(-13.1 to 5.4) | -1.5(-7.9 to 2.9) |
|  | **FOS** | 6 | 20.4(9.5-31.4) | 28.0(15.9-40.0) | - | 0.0(0.0-13.5) | 8.3(2.4-14.2) | - | - |
| **Not in AWaRe** | **IMP** | 15 | 2.8(1.0-4.6) | 4.3(0.7-7.8) | 1.8(0.0-7.4) | 15.6(0.0-35.0) | 0.5(0.0-1.70 | -0.8(-4.2 to 2.6) | -0.7(-2.7 to 1.3) |
|  | **NA** | 19 | 42.5(26.4-58.5) | 82.8(73.5-92.2) | 28.6(20.5-36.7) | 30.7(13.3 to 48.2) | 19.5(10.1-29.0) | 5.7(-2.9 to 14.5) | -20.1(-25.4 to -16.3) |
|  | **DAN** | 5 | 40.1(1.1-79.0) | 63.2(52.7-73.6) | - | 11.1(0.0-31.6) | 0.0(0.0-0.5) | - | - |
|  | **LS** | 9 | 9.4(5.5-13.4) | 2.3(8.7-39.9) | - | 0.0(0.0-1.4) | 5.4(0.0-12.7) | - | - |
|  | **SUL** | 6 | 26.0(13.2-38.8) | 54.9(44.6-65.3) | 47.1(38.1-56.0) | - | 2.2(0.0-5.8) | - | - |
|  | **FFC** | 12 | 33.3(20.6-46.0) | 53.2(31.7-74.8) | - | 42.5(17.1-68.0) | 4.4(0.0-8.7) | 11.9(0.3 to 23.6) | -14.9(-21.7 to -8.1) |

**Figure S6 Significant AMR trend of E. coli isolated from animal samples during time-points**

**Antibiotic resistance in Environmental samples**

Environmental samples include food and environmental samples. The prevalence of AMR was investigated on different types of samples like to beef and chicken meat, egg, Raw milk, Vegetable, traditional milk products, processed meat products, fruit, restaurant salads, ready to-eat foods (sausage), ICU environment, tracheal tubes, used medical mask, urinary catheters, river and spring water, wastewater, different fish fields, etc.

**Table S6 Pooled prevalence of AMR of E. coli and meta-regression (between group and during time-point) in environmental samples, 2010-2025, Iran.**

|  | **Abbreviations** | **No of study** | **Total** | **Animal produces** | **Vegetable** | **Medical tools** | **Wastewater/water** | **Time-point** | **Group** |
| --- | --- | --- | --- | --- | --- | --- | --- | --- | --- |
| **Access** | AMC | 11 | 61.7(33.1-90.3) | 73.3(31.5-115.2) | 25.0(0.0-67.4) | 100.0(67.6-132.4) | 38.0(0.0-80.3) | -27.5(-59.1 to 4.2) | -19.0(-36.8 to -1.1) |
|  | AN | 23 | 26.1(19.5-32.7) | 30.7(19.1-42.4) | 19.3(0.0-58.4) | 39.2(15.1-63.2) | 12.4(3.8-21.1) | -12.7(-21.9 to -3.5) | -5.8(-11.9 to 0.2) |
|  | AMX | 8 | 71.8(49.4-94.2) | 81.1(53.5-100.0) | 50.0(1.0-99.0) | - | 51.1(11.3-90.8) | - | - |
|  | AM | 28 | 63.7(52.2-75.2) | 70.5(56.2-84.8) | 74.5(50.8-98.2) | 35.6(0.0-82.1) | 53.9(22.1-85.6) | -21.0(-34.1 to -7.5) | -4.2(-12.9 to 4.4) |
|  | C | 25 | 30.5(17.6-43.4) | 28.9(18.7-39.2) | 25.3(11.0-39.6) | 27.5(13.7-41.3) | 36.6(0.0-98.7) | 4.8(-13.4 to 23.1) | 0.9(-10.1 to 11.9) |
|  | CLT | 7 | 39.5(2.4-76.6) | 35.8(0.0-90.4) | 32.8(25.9-39.6) | - | 50.3(0.0-100.0) | - | - |
|  | CLX | 3 | 42.2(0.0-100.0) | 42.2(0.0-100.0) | - | - | - | - | - |
|  | CZ | 2 | 86.1(59.1-100.0) | 100.0(85.0-100.0) | - | 72.5(58.7-86.3) | - | - | - |
|  | DO | 3 | 61.0(21.2-100.0) | 80.0(69.4-90.6) | - | - | 25.0(6.0-44.0) | - | - |
|  | FM | 11 | 37.0(19.5-54.6) | 39.9(4.6-75.2) | 42.4(0.0-97.9) | 53.5(41.9-65.1) | 16.2(8.0-24.4) | 34.2(-0.7 to 69.1) | -1.1(-14.6 to 12.2) |
|  | GM | 34 | 38.3(27.6-48.9) | 41.6(28.3-54.9) | 57.1(21.7-92.6) | 36.7(15.8-57.6) | 19.1(0.0-42.0) | -27.3(-43.8 to -10.8) | -4.5(-14.5 to 5.4) |
|  | P | 5 | 55.4(27.2-83.6) | 70.4(52.9-87.9) | - | - | 0.0(0.0-6.5) | - | - |
|  | SAM | 2 | 49.7(0.0-100.0) | 49.7(0.0-100.0) | - | - | - | - | - |
|  | SMX | 9 | 44.6(25.9-63.3) | 46.6(26.1-67.2) | 70.0(41.6-98.4) | 0.0(0.0-32.4) | - | - | - |
|  | SXT | 25 | 45.5(23.9-67.1) | 46.3(19.0-73.6) | 69.1(32.5-100.0) | 52.3(30.8-73.8) | 33.2(14.5-51.8) | -7.8(-47.0 to 31.3) | -4.2(-24.7 to 16.4) |
|  | TE | 32 | 67.3(50.0-84.5) | 74.2(49.0-99.5) | 71.1(52.7-89.5) | 68.7(52.2-85.3) | 47.8(7.6-88.0) | -9.1(-33.7 to 15.6) | -8.3(-22.2 to 5.7) |
|  | TMP | 11 | 38.2(23.4-53.1) | 33.4(20.0-46.8) | 45.3(38.2-52.3) | - | - | -7.7(-47.2 to 32.0) | 4.2(-16.3 to 24.7) |
| **Watch** | AZM | 8 | 28.2(6.1-50.3) | 33.3(4.8-61.8) | 14.6(0.0-34.5) | - | - | - | - |
|  | CAZ | 18 | 38.0(24.9-51.0) | 36.9(20.8-53.1) | 20.9(0.0-51.0) | 52.8(44.6-61.0) | 39.5(0.0-100.0) | -6.7(-24.5 to 11.1) | -1.6(-13.0 to 9.9) |
|  | CFM | 5 | 23.7(12.2-35.1) | 19.7(9.3-30.0) | - | 42.3(30.8-53.7) | 15.0(0.0-30.6) | - | - |
|  | CIP | 33 | 48.9(36.3-61.4) | 49.8(36.2-63.3) | 45.8(13.8-77.8) | 58.0(41.7-74.3) | 42.9(11.3-74.5) | -9.7(-27.7 to 8.4) | -1.9(-13.2 to 9.3) |
|  | CRO | 8 | 38.2(12.7-63.8) | 30.2(2.1-58.4) | - | 61.9(42.0-81.9) | - | - | - |
|  | CTX | 18 | 35.0(23.4-46.6) | 41.9(27.6-56.2) | 28.4(15.9-40.9) | 37.8(0.0-100.0) | 8.7(0.0-18.0) | -1.6(-17.5 to 14.3) | -9.1(-17.0 to -1.2) |
|  | E | 6 | 46.2(13.3-79.1) | 26.0(0.0-52.1) | 73.2(64.4-82.0) | 54.9(45.2-64.6) | - | - | - |
|  | ENR | 11 | 42.2(29.9-54.6) | 46.2(29.5-63.0) | 36.9(0.0-76.2) | - | - | 21.7(-2.1 to 45.5) | -2.1(-18.5 to 14.4) |
|  | FEP | 10 | 22.3(0.9-43.6) | 28.0(4.0-52.1) | 25.0(0.0-67.4) | 0.0(0.0-32.4) | 0.0(0.0-2.9) | -1.3(-40.4 to 40.1) | -8.3(-40.0 to 15.2) |
|  | FOX | 8 | 16.2(8.5-23.8) | 15.0(4.5-25.6) | 21.8(13.7-29.8) | 0.0(0.0-32.4) | - | - | - |
|  | K | 6 | 35.1(0.0-75.7) | 42.1(0.0-87.1) | 0.0(0.0-26.3) | - | - | - | - |
|  | LVX | 5 | 18.5(0.0-39.3)) | 22.8(0.0-56.6) | 0.0(0.0-26.3) | - | 19.6(8.7-30.5) | - | - |
|  | MEM | 4 | 0.0(0.0-2.9) | 3.7(0.0-16.4) | 0.0(0.0-1.4) | - | 0.0(0.0-2.9) | - | - |
|  | NX | 7 | 37.2(6.2-68.1) | 11.8(0.0-35.0) | 0.0(0.0-26.3) | 100.0(67.6-100.0) | 59.4(1.6-100.0) | - | - |
|  | PIP | 3 | 49.7(17.7-81.7) | - | - | 29.6(0.0-83.3) | 81.7(71.9-91.5) | - | - |
|  | STR | 16 | 36.9(11.2-62.6) | 28.2(17.0-39.3) | 25.8(4.6-47.1) | - | 56.8(0.0-100.0) | 19.3(0.9 to 37.6) | 5.4(-5.1 to 15.8) |
|  | VA | 4 | 41.0(0.0-100.0) | 54.2(0.0-100.0) | - | 0.0(0.0-32.4) | - | - | - |
| **Reserve** | ATM | 4 | 56.8(25.6-88.0) | 45.9(0.0-100.0) | - | 50.0(34.5-65.5) | 85.0(76.0-94.0) | - | - |
|  | CL | 4 | 24.6(0.0-55.2) | 33.4(0.0-73.4) | 0.0(0.0-26.3) | - | - | - | - |
|  | PB | 2 | 22.5(0.0-71.2) | 22.5(0.0-71.2) | - | - | - | - | - |
| **Not in AWaRe** | IMP | 27 | 15.4(10.6-20.2) | 12.0(5.9-18.2) | 10.1(2.9-17.3) | 34.3(15.2-53.5) | 12.6(4.5-20.7) | -5.1(-11.7 to 1.6) | -0.3(-4.4,3.7) |
|  | NA | 13 | 37.2(0.0-90.3) | 47.8(2.4-93.3) | 0.0(0.0-26.3) | 67.6(56.7-78.5) | 27.4(5.5-49.3) | -8.1(-37.9 to 21.6) | -0.9(-24.9 to 6.7) |
|  | AOZ | 3 | 34.9(16.6-53.2) | 41.8(28.9-54.8) | - | - | 20.0(2.5-37.5) | - | - |

**Figure S7Significant AMR trend of E. coli isolated from environmental samples during time-points.**

**Results of ESBL and β-lactamase ARG**

**Table S7 The prevalence of ESBL isolates of E. coli and β-lactamase ARGs distribution in different provinces.**

| **Province** | **ESBL** | | **TEM** | | **SHV** | | **CTXM** | |
| --- | --- | --- | --- | --- | --- | --- | --- | --- |
|  | **No. of Study** | **Prevalence (95% CI)** | **No. of Study** | **Prevalence (95% CI)** | **No. of Study** | **Prevalence (95% CI)** | **No. of Study** | **Prevalence (95% CI)** |
| **Alborz** | 6 | 40.0 (22.7-57.2) | 1 | 56.0 (45.3-66.6) | 1 | 64.3 (54.0-74.5) | 1 | 21.4 (12.7-30.2) |
| **Ardabil** | 1 | 46.6 (39.0-54.3) |  |  |  |  |  |  |
| **Chaharmahal and Bakhtiari** | 1 | 45.0 (38.1-51.9) |  |  |  |  |  |  |
| **East Azerbaijan** | 4 | 39.1 (14.8-63.4) |  |  |  |  |  |  |
| **Fars** | 16 | 42.8 (30.2-55.4) | 5 | 52.7 (27.1-78.3) | 5 | 23.7 (1.0-46.4) | 5 | 75.5 (57.9-93.0) |
| **Guilan** | 11 | 43.4 (27.0-59.8) | 3 | 41.4 (34.2-48.6) | 3 | 53.2 (29.2-77.1) | 3 | 42.9 (28.4-57.4) |
| **Hamedan** | 1 | 34.8 (26.7-43.0) | 1 | 66.1 (53.7-78.5) | 1 | 32.1 (19.9-44.4) | 1 | 1.8 (0.0-5.3) |
| **Ilam** | 3 | 62.6 (51.3-73.9) | 2 | 84.0 (59.7-100.0) | 3 | 5.4 (0.0-12.6) | 2 | 82.8 (55.7-100.0) |
| **Isfahan** | 7 | 43.4 (32.9-53.9) |  |  |  |  | 1 | 82.9 (70.4-95.3) |
| **Kerman** | 5 | 36.0 (23.8-48.2) | 3 | 74.3 (68.6-80.0) | 3 | 1.5 (0.0-3.1) | 2 | 65.6 (47.4-83.9) |
| **Kermanshah** | 6 | 32.4 (20.5-44.3) | 1 | 37.5 (18.1-56.9) | 1 | 4.2 (0.0-12.2) | 3 | 77.1 (55.5-98.7) |
| **Khuzestan** | 12 | 64.9 (53.9-76.0) | 7 | 50.0 (35.9-64.1) | 5 | 13.4 (0.0-33.3) | 7 | 73.7 (60.3-87.0) |
| **Kohgiluyeh and Boyer-Ahmad** | 1 | 43.0 (36.1-49.9) |  |  |  |  | 1 | 95.3 (90.9-99.8) |
| **Kurdistan** | 1 | 22.3 (15.2-29.5) | 1 | 66.7 (28.9-100.0) | 1 | 0.0 (0.0-19.1) |  |  |
| **Lorestan** | 3 | 47.6 (11.2-84.0) |  |  |  |  | 2 | 61.6 (29.2-93.9) |
| **Markazi** | 4 | 56.5 (48.3-64.8) | 2 | 100.0 (94.1-100.0) | 2 | 33.3 (16.5-50.2) | 2 | 90.7 (80.8-100.0) |
| **Mazandaran** | 6 | 52.6 (33.3-71.8) | 2 | 47.7 (40.7-54.7) | 1 | 32.0 (22.7-41.2) | 2 | 20.7 (11.3-30.0) |
| **Qazvin** | 2 | 32.9 (24.9-41.0) |  |  |  |  |  |  |
| **Qom** | 2 | 53.2 (46.7-59.7) | 1 | 54.2 (41.5-66.9) | 1 | 15.3 (6.1-24.4) | 1 | 88.1 (79.9-96.4) |
| **Khorasan Razavi** | 6 | 53.6 (33.3-73.8) | 4 | 56.8 (43.7-70.0) | 3 | 11.2 (0.0-23.3) | 3 | 76.3 (44.5-100.0) |
| **Semnan** | 4 | 55.7 (15.2-96.2) |  |  |  |  | 1 | 69.0 (52.1-85.8) |
| **Sistan and Baluchistan** | 2 | 65.3 (52.2-78.5) | 1 | 53.6 (40.5-66.6) | 1 | 19.6 (9.2-30.0) | 1 | 85.7 (76.5-94.9) |
| **South Khorasan** | 2 | 57.3 (0.0-100.0) | 1 | 88.9 (74.4-100.0) | 1 | 66.7 (44.9-88.4) | 1 | 100.0 (92.8-100.0) |
| **Tehran** | 25 | 43.5 (35.2-51.8) | 8 | 56.5 (43.3-69.6) | 8 | 31.4 (12.2-50.6) | 11 | 78.6 (70.4-86.7) |
| **West Azerbaijan** | 1 | 0.0 (-2.4-2.4) |  |  |  |  | 1 | 75.8 (65.4-86.1) |
| **Zanjan** | 1 | 33.0 (26.5-39.5) | 1 | 47.0 (34.9-59.0) | 1 | 56.1 (44.1-68.0) | 1 | 25.0 (0.0-67.4) |

**Table S8 pooled prevalence of AMR and 95% CI for ESBL-positive and ESBL-negative E. coli isolates, 2010-2025, Iran.**

| **AWaRe** | **Antibiotics** |  | **ESBL -** |  | **ESBL+** |  |
| --- | --- | --- | --- | --- | --- | --- |
|  |  | **No. of Study** | **Pooled prevalence (95% CI)** | **No. of Study** | **Pooled prevalence (95% CI)** | **Meta-regressionBetween groups** |
| **Access** | **Amoxicillin / Clavulanic acid** | 9 | 59.0(38.3-79.7) | 12 | 69.4 (56.5-82.2) | -10.1(-32.8 to 12.5) |
|  | **Amikacin** | 13 | 10.5(6.8-14.3) | 29 | 20.4(10.2-30.6) | -3.7(-8.9 to 1.5) |
|  | **Amoxicillin** | 5 | 63.6 (29.1-98.1) | 9 | 76.8 (66.4-87.2) | -12.6 (-34.7 to 9.5) |
|  | **Ampicillin** | 14 | 74.0 (63.8-84.2) | 19 | 82.5 (71.4-93.5) | -8.6 (-25.3to 8.1) |
|  | **Chloramphenicol** | 4 | 30.4 (17.0-43.8) | 9.0 | 30.3(6.5-54.1) | -1.2 (-38.5 to 41.0) |
|  | **Cephalothin** | 2 | 50.9 (0.00-100.0) | 2.0 | 80.5 (41.9- 100.0) | - |
|  | **Cephalexin** | 4 | 53.5 (43.1- 63.8) | 8.0 | 86.6 (81.0- 92.1) | -33.0 (-43.5 to -22.5) |
|  | **Cefazolin** | 5 | 48.1(23.3-73.0) | 6 | 90.6(84.0-97.2) | -40.8(-58.2to-23.5) |
|  | **Cefpodoxime** | 1 | 53.0(41.0-65.1) | 1 | 73.5(58.7-88.4) |  |
|  | **Nitrofurantoin** | 10 | 10.1(5.5-14.8) | 21 | 21.7(6.4-37.0) | -10.2(-32.2to11.8) |
|  | **Gentamicin** | 19 | 15.2(11.3-19.1) | 35 | 42.2(36.9-47.5) | -26.2(-33.1to-19.3) |
|  | **Penicillin** | 1 | 100.0 (97.3-100.0) | 3 | 100.0 (98.9- 100.0) | - |
|  | **Ampicillin/Sulbactam** | 2 | 25.2(0.0-64.9) | 5 | 51.7(35.6-67.8) | - |
|  | **Trimethoprim- Sulfamethoxazole** | 17 | 50.7(45.9-55.4) | 32 | 75.8(69.7-81.8) | -25.2(-34.9to-15.5) |
|  | **Tetracycline** | 7 | 57.8 (46.9- 68.6) | 9 | 72.0(60.1-83.9) | -14.1(-31.0to-2.5) |
|  | **Sulfadiazine/trimethoprim** | 1 | 52.1( 42.0- 62.2) | 2 | 90.8 ( 79.1- 100.0) | - |
| **Watch** | **Ceftazidime** | 17 | 15.1 ( 10.6- 19.7) | 34 | 69.8(62.6-77.0) | -53.0(-63.0to-43.0) |
|  | **Cefixime** | 9 | 29.9 ( 15.0- 44.9) | 13 | 94.7 ( 92.0- 97.3) | -65.0(-72.0to-58.0) |
|  | **Ciprofloxacin** | 20 | 35.1 ( 28.7-41.6) | 36 | 64.7(57.5to72.0) | -29.0(-40.0to-18.0) |
|  | **Ceftriaxone** | 15 | 22.8(14.5-31.1) | 23 | 87.7(84.0-91.4) | -65.8(-73.0to-58.0) |
|  | **Cefuroxime** | 6 | 47.5(13.2-81.8) | 9 | 79.6(58.2to100.0) | -32.2(-66.3to2.1) |
|  | **Cefotaxime** | 15 | 20.3(13.1- 27.6) | 28 | 81.1(75.6-86.7) | -61.0(-70.1to-51.4) |
|  | **Ceftizoxime** | 1 | 2.1 (0.0-6.1) | 2 | 57.9 ( 34.3- 81.6) | - |
|  | **Ertapenem** | 4 | 1.1 (0.0-3.4) | 4 | 1.6 (0.0- 4.3) | - |
|  | **Cefepime** | 8 | 12.6 (6.4-18.9) | 15 | 64.8(56.8-72.8) | -51.3(-62.0to-40.7) |
|  | **Cefoxitin** | 5 | 13.0 ( 7.5-18.4) | 9 | 33.8(7.3-60.3) | -19.2(-55.5to17.1) |
|  | **Levofloxacin** | 5 | 25.0(9.1-41.0) | 7 | 53.1(24.0-82.2) | -27.6(-63.0to7.9) |
|  | **Meropenem** | 8 | 1.7(0.3-3.2) | 14 | 13.8(0.0-43.5) | -10.8(-49.4to27.8) |
|  | **Norfloxacin** | 4 | 27.7 ( 22.6- 32.8) | 4 | 60.8 (38.5- 83.2) | - |
|  | **Ofloxacin** | 1 | 15.1(6.9-23.3) | 1 | 48.1(29.3-67.0) | - |
|  | **Piperacillin** | 4 | 32.1(6.9-57.3) | 7 | 40.7(1.6-79.8) | -6.4(-38.6to25.7) |
|  | **Streptomycin** | 2 | 39.2(0.0-80.5)_ | 4 | 65.1(51.8-78.5) | - |
|  | **Tobramycin** | 3 | 14.8(3.9-25.7) | 5 | 55.0(30.1-79.9) | - |
|  | **Ticarcilin** | **1** | 70.8 (58.0-83.7) | 2 | 93.6 (80.5-100.0) | - |
|  | **Piperacillin- Tazobactam** | 6 | 5.3(1.0-9.6) | 12 | 16.5(9.7-23.2) | -3.2(-7.5to1.2) |
|  | **Colistin** | 1 | 2.1(0.0-35.1) | 4 | 32.7(13.6-51.9) | - |
|  | **Fosfomycin** | 2 | 25.0(0.0-63.1) | 3 | 22.6 (0.0-47.5) | - |
|  | **Polymyxin-B** | 1 | 2.1(0.0-6.1) | 1 | 0.0(0.0-2.0) | - |
| **Not in AWaRe** | **Imipenem** | 16 | 3.8(1.8-5.7) | 31 | 16.2(10.6-21.7) | -7.4(-13.2to-1.7) |
|  | **Nalidixic acid** | 8 | 55.1(47.5-63.3) | 16 | 74.8(67.1-82.4) | -20.0(-32.8to-7.3) |

**Table S 9 Prevalence of β-lactamase ARGs in ESBL, AmpC, and MBL-producing isolates of E. coli, 2010-2025, Iran.**

| **Groups** | **Genes** | No.  **of studies** | No  **of isolates** | Pooled prevalence | 95% CI | I^2^ | Meta regression |
| --- | --- | --- | --- | --- | --- | --- | --- |
| **ESBL+** | *bla_TEM_* | 40 | 2853 | 58.5 | 51.9-65.4 | 93.6 | -4.1(-13.1 to 4.9) |
|  | *bla_SHV_* | 42 | 2630 | 24.4 | 18.0-30.8 | 97.5 | -8.3(-17.5 to 0.8) |
|  | *bla_CTX M_* | 52 | 3609 | 68.2 | 56.4-79.9 | 99.27 | -0.1 (-0.2 to 0.1) |
|  | *bla_CTX M1_* | 10 | 557 | 73.8 | 57.6-89.9 | 95.9 | 6.0 (-28.4 to 40.3) |
|  | *bla_CTX M2_* | 5 | 296 | 13.8 | 0.1- 27.4 | 89.5 | - |
|  | *bla_CTX M3_* | 2 | 206 | 10.7 | 0.0- 33.5 | 92.3 | - |
|  | *bla_CTX M8_* | 3 | 124 | 0.0 | 0.0- 1.4 | 0.0 | - |
|  | *bla_CTX M9_* | 3 | 174 | 29.9 | 0.0- 67.3 | 97.3 | - |
|  | *bla_CTX M14_* | 4 | 140 | 44.9 | 28.9- 60.8 | 70.6 | - |
|  | *bla_CTX M15_* | 10 | 605 | 84.4 | 76.4-92.5 | 86.9 | 8.6 (-8.7 to 26.0) |
|  | *bla_CTX M_ + bla_TEM_* | 19 | 1154 | 23.3 | 16.6-30.0 | 93.6 | -2.4(-12.6 to 7.8) |
|  | *bla_CTX M_ + bla_TEM_ + bla_SHV_* | 13 | 824 | 9.2 | 5.2-13.1 | 80.9 | 4.1(-0.8 to 9.1) |
|  | *bla_CTX M_ + bla_SHV_* | 13 | 947 | 7.6 | 4.5-10.8 | 87.2 | -3.3(-8.5 to 1.9) |
|  | *bla_TEM_+bla_SHV_* | 15 | 995 | 8.3 | 4.6- 12.0 | 85 | -4.7(-9.3 to -0.2) |
|  | *bla_OXA_* | 6 | 648 | 20.1 | 5.1- 35.1 | 97.6 | - |
|  | *bla_OXA48_* | 4 | 244 | 8.1 | 1- 15.1 | 86.4 | - |
|  | *bla_PER_* | 5 | 422 | 0.1 | 0.1-0.8 | 1.5 | - |
|  | *bla_VEB_* | 4 | 352 | 2.7 | 0.0- 5.9 | 80 | - |
| **AMPC** | *bla_NDM_* | 2 | 35 | 4.1 | 0.0-10.6 |  |  |
|  | *bla_ACC_* | 5 | 96 | 30.5 | 0.0-64.5 | 96.1 |  |
|  | *bla_FOX_* | 3 | 49 | 6.2 | 0.0-14.1 | 2.74 |  |
|  | *bla_MOX_* | 4 | 97 | 1.7 | 0.0-5.1 | 58.56 |  |
|  | *bla_DHA_* | 6 | 126 | 18.9 | 1.2-25.1 | 69.1 |  |
|  | *bla_CIT_* | 5 | 115 | 62.2 | 28.4-95.9 | 94.9 |  |
|  | *bla_EBC_* | 2 | 83 | 10.8 | 4.2-17.4 | - |  |
|  | *bla_CMY_* | 6 | 97 | 44.9 | 20.2-69.7 | 86.6 |  |
| **MBL** | *bla_VIM_* | 4 | 81 | 2.4 | 0.0- 6.1 | 35.2 | - |
|  | *bla_IMP_* | 5 | 81 | 22.8 | 0.0-53.7 | 93.4 | - |
|  | *bla_NDM_* | 3 | 76 | 30.1 | 1.4-58.9 | 91.9 | - |
|  | *bla_OXA 48_* | 2 | 60 | 5.6 | 01-11.2 |  |  |
|  | *bla_OXA 23_* | 2 | 60 | 1.8 | 0.5-5.6 |  |  |

**Table S10 Studies with reporting the standard multilocus sequence typing (MLST) scheme**

| **Reference** | **allelic profile** | | **Methods** | **Sequence Type** | **Article** |
| --- | --- | --- | --- | --- | --- |
| **(1)** | 103 | *adk* | Achtman | 472 | Considerable rate of putative virulent phylo-groups in fecal carriage of extended-spectrum β-lactamase producing *Escherichia coli* |
|  | 126 | *fum*C |  |  |  |
|  | 5 | *gyr*B |  |  |  |
|  | 1 | *icd* |  |  |  |
|  | 8 | *mdh* |  |  |  |
|  | 8 | *pur*A |  |  |  |
|  | 6 | *rec*A |  |  |  |
| **(2)** | 2 | *din*B | Pasteur | 131 | High prevalence of sequence type 131 isolates producing CTX-M-15 among extended-spectrum β-lactamase-producing *Escherichia coli* strains in northeast Iran |
|  | 65 | *icd*A |  |  |  |
|  | 3 | *pab*B |  |  |  |
|  | 10 | *pol*B |  |  |  |
|  | 26 | *put*P |  |  |  |
|  | 7 | *trp*A |  |  |  |
|  | 4 | *trp*B |  |  |  |
|  | 57 | *uid*A |  |  |  |
| **(3)** | 3 | *din*B | Pasteur | 77 | Characterization of antimicrobial susceptibility, extended-spectrum β-lactamase genes and phylogenetic groups of Shigatoxin producing *Escherichia coli* isolated from patients with diarrhea in Iran |
|  | 43 | *icd*A |  |  |  |
|  | 31 | *pab*B |  |  |  |
|  | 33 | *pol*B |  |  |  |
|  | 8 | *put*P |  |  |  |
|  | 3 | *trp*A |  |  |  |
|  | 5 | *trp*B |  |  |  |
|  | 11 | *uid*A |  |  |  |
|  | 25 | *din*B | Pasteur | 1007 |  |
|  | 3 | *icd*A |  |  |  |
|  | 4 | *pab*B |  |  |  |
|  | 146 | *pol*B |  |  |  |
|  | 78 | *put*P |  |  |  |
|  | 29 | *trp*A |  |  |  |
|  | 2 | *trp*B |  |  |  |
|  | 5 | *uid*A |  |  |  |
|  | 9 | *din*B | Pasteur | 506 |  |
|  | 134 | *icd*A |  |  |  |
|  | 74 | *pab*B |  |  |  |
|  | 134 | *pol*B |  |  |  |
|  | 4 | *put*P |  |  |  |
|  | 72 | *trp*A |  |  |  |
|  | 1 | *trp*B |  |  |  |
|  | 9 | *uid*A |  |  |  |

**Table S11 IDSA Guidelines of antibiotics for the treatment of ESBL-producing Enterobacterales disease, and the prevalence of AMR in the Present study.**

| **Indication** | **Antibiotic** | **IDSA suggestion** | **Prevalence in this study** | | |
| --- | --- | --- | --- | --- | --- |
|  |  |  | **Total isolates** | **Total humans** | **ESBL(+) isolates** |
| **Uncomplicated cystitis caused by ESBL-E** | Nitrofurantoin | Preferred | 13.3 | 11.2 | 21.7 |
|  | SXT | Preferred | 54.0 | 57.9 | 90.8 |
|  | Ciprofloxacin | Alternative | 41.4 | 41.5 | 64.7 |
|  | Levofloxacin | Alternative | 34.5 | 38.3 | 53.1 |
|  | Imipenem | Alternative | 11.9 | 11.8 | 16.2 |
|  | Ertapenem | Alternative | 4.7 | 4.9 | 1.6 |
|  | Meropenem | Alternative | 7.3 | 8.0 | 13.8 |
|  | Doripenem | Alternative | 3.8 | 57.4 | - |
|  | Amikacin | Alternative | 14.8 | 16.8 | 20.4 |
|  | Gentamicin | Alternative | 27.5 | 29.0 | 42.2 |
|  | Streptomycin | Alternative | 14.2 | 33.4 | 65.1 |
|  | Kanamycin | Alternative | 22.9 | 18.1 | - |
|  | Tobramycin | Alternative | 33.5 | - | 55.0 |
|  | Fosfomycin | Alternative | 8.0 | 6.3 | 22.6 |
| **Pyelonephritis or cUTIs** | SXT | Preferred | 54.0 | 57.9 | 90.8 |
|  | Ciprofloxacin | Preferred | 41.4 | 41.5 | 64.7 |
|  | Levofloxacin | Preferred | 34.5 | 38.3 | 53.1 |
|  | Ertapenem | Preferred* | 4.7 | 4.9 | 1.6 |
|  | Meropenem | Preferred* | 7.3 | 8.0 | 13.8 |
|  | Doripenem | Preferred* | 3.8 | 57.4 | - |
|  | Amikacin | Alternative | 14.8 | 16.8 | 20.4 |
|  | Gentamicin | Alternative | 27.5 | 29 | 42.2 |
|  | Streptomycin | Alternative | 14.2 | 33.4 | 65.1 |
|  | Kanamycin | Alternative | 22.9 | 18.1 | - |
|  | Tobramycin | Alternative | 33.5 | - | 55 |
| **Infections outside of the urinary tract** | Meropenem | Preferred | 7.3 | 8.0 | 13.8 |
|  | Ertapenem | Preferred | 4.7 | 4.9 | 1.6 |

*The preferred antibiotics for the treatment of uncomplicated cystitis, pyelonephritis, or complicated UTIs (cUTIs), and infections outside of the urinary tract caused by ESBL-producing Enterobacterales based on IDSA 2024 Guidance. The prevalence of AMR for these antibiotics in total isolates, based on Table 1, prevalence in human Isolates, based on Table S4, and prevalence in ESBL-p isolates, based on Table S8.*** Ertapenem, meropenem, and imipenem-cilastatin are preferred agents when resistance or toxicities preclude the use of TMP-SMX or fluoroquinolones.**
